# Integrative Network Analysis of Differentially Methylated and Expressed Genes for Biomarker Identification in Leukemia

**DOI:** 10.1038/s41598-020-58123-2

**Published:** 2020-02-07

**Authors:** Robersy Sanchez, Sally A. Mackenzie

**Affiliations:** 1 Department of Biology, The Pennsylvania State University, University Park, PA 16802 USA; 2 Department of Plant Science, The Pennsylvania State University, University Park, PA 16802 USA

**Keywords:** Cancer genomics, Diagnostic markers

## Abstract

Genome-wide DNA methylation and gene expression are commonly altered in pediatric acute lymphoblastic leukemia (PALL). Integrated network analysis of cytosine methylation and expression datasets has the potential to provide deeper insights into the complex disease states and their causes than individual disconnected analyses. With the purpose of identifying reliable cancer-associated methylation signal in gene regions from leukemia patients, we present an integrative network analysis of differentially methylated (DMGs) and differentially expressed genes (DEGs). The application of a novel signal detection-machine learning approach to methylation analysis of whole genome bisulfite sequencing (WGBS) data permitted a high level of methylation signal resolution in cancer-associated genes and pathways. This integrative network analysis approach revealed that gene expression and methylation consistently targeted the same gene pathways relevant to cancer: *Pathways in cancer, Ras signaling pathway*, *PI3K-Akt signaling pathway*, and *Rap1 signaling pathway*, among others. Detected gene hubs and hub sub-networks were integrated by signature loci associated with cancer that include, for example, *NOTCH1, RAC1, PIK3CD, BCL2*, and *EGFR*. Statistical analysis disclosed a stochastic deterministic relationship between methylation and gene expression within the set of genes simultaneously identified as DEGs and DMGs, where larger values of gene expression changes were probabilistically associated with larger values of methylation changes. Concordance analysis of the overlap between enriched pathways in DEG and DMG datasets revealed statistically significant agreement between gene expression and methylation changes. These results support the potential identification of reliable and stable methylation biomarkers at genes for cancer diagnosis and prognosis.

## Introduction

Network-based modeling approaches have the potential to integrate and improve the perception of complex disease states and their root causes. To date, network analysis provides reliable and cost effective approaches for early disease detection, prediction of co-occurring diseases and interactions, and drug design^1^. Although integrated genomic analysis of methylation and gene expression in leukemia has been reported^2–5^, meaningful assimilation of network analysis is still lacking.

Our study investigates protein-protein interaction networks (PPI), which are exclusively focused on protein-protein associations and resulting cell activities. A PPI network can be defined as a (un)directed graph/network holding vertices as proteins (or protein-coding genes) and edges as the interactions/association between them. Associations are meant to be specific and biologically meaningful, i.e., two proteins are connected by an edge if jointly contributing to a shared function, which does not necessarily reflect a physical binding interaction.

Within the network, some proteins denote hubs interacting with numerous partners. Biologically, hubs are key elements on which functionality of the cellular process modeled by the network depends. A significant report on the vulnerability of network hubs from p53 protein interaction network, which plays a critical role in the progression of several cancer types, was made by Dartnell *et al*.^6^. Although inherently robust to random knockouts of its proteins, this network is vulnerable to the loss of its hubs, which leads to the disruption of cell cycle and apoptosis systems. Numerous studies on protein networks show that deleting a highly connected protein node (hub) is more likely to be lethal to an organism than deleting a low connection node (non-hub)^6–8^. This observation reflects the centrality–lethality rule, where high-degree proteins or hubs tend to be more essential than low-degree proteins. Consequently, it is reasonable to assume that a biomarker suitable to define specific disease states would likely be a hub or a hub regulator within a relevant network. Frequently, more than one interacting network model is possible, with each model carrying a different uncertainty level for the biological process under study. Integration of more than one network model can help to reduce the implicit uncertainty associated to each model prediction^6^.

Integrative network analysis of cytosine DNA methylation and gene expression data in patients with cancer has resulted in several published reports^9–13^, typically with data from The Cancer Genome Atlas (TCGA). Yet, studies have not capitalized on whole genome bisulfite sequence (*WGBS*) data, which offers greater resolution. Here we focus on Leukemia.

We report on integrative network analysis of DMGs, DEGs and DEG-DMGs, where the identification of DMGs was accomplished with the application of a novel signal detection-machine learning approach, Methyl-IT^14^, on WGBS data. We show that integrating Methyl-IT results with gene expression analysis in a network context permits greater resolution for cancer-associated genes and pathways than observed previously.

We address the hypothesis that disease-induced DNA methylation changes can serve as a source of reliable and stable biomarkers for cancer. Toward that aim, aberrant DNA methylation of key genes was reported in Acute Lymphoblastic Leukemia (ALL)^15^, and we have tested a reproducible approach to integrating network analysis of DMGs, DEGs and DEG-DMGs within datasets from patients with pediatric ALL (PALL). This data integration may provide the basis for robust identification of reliable and stable biomarkers.

## Results

**Figure 1:**
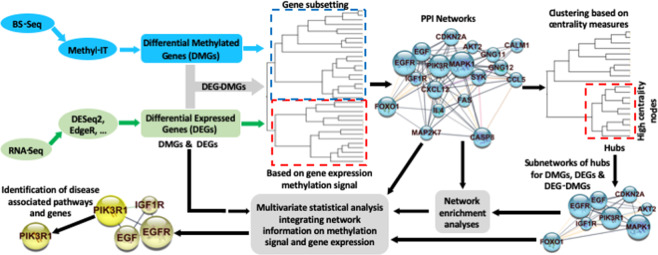
Flow chart of the analyses used in the current study. Initial data are from RNAseq and BS-seq experiments. Methyl-IT^14^ facilitates integrative analysis of methylation and gene expression data. Once DMGs, DEGs and DEG-DMGs are identified, different analytical/algorithmic approaches (i.e., cluster analysis or Bayesian networks^11^) can be applied to objectively extract biologically meaningful subsets of genes for STRING PPI-network identification^16^. Network enrichment analyses are performed on the PPI networks and subnetworks. The latter are generally detected with clustering approaches based on network centrality measures. Multivariate statistical analyses can be accomplished with DMGs, DEGs and DEG-DMGs at any step. For example, direct interaction between PIK3R and EGFR, detected in our analyses, and the role of PIK3R in cancer are validated in the BioGRID^17^ database (https://thebiogrid.org/).

The flow chart presented in Fig. 1 summarizes the relevant steps of the procedure followed in our study. While the general workflow is consistent with current developments in network analysis, enhanced resolution is pursued with the application of Methyl-IT analysis.

### General methylation features of the study

The distribution of methylation changes at DMPs along the chromosome revealed genome-wide methylation repatterning dominated by hypermethylation in PALL patients (Supplementary Fig. S1). Hypomethylated sites are visible in the genome browser after zooming (tracks available in the Supplementary File S1). Consistent with natural methylation variability in the population of healthy individuals, DMPs were observed in the control group as well.

Methyl-IT analysis yielded a total of 4795 DMGs, including protein-coding regions (3338) and non-coding RNA genes (Supplementary Table S1). 1774 genes from the set of 2360 (B cell) DEGs reported in the original study^18^ were DMGs here as well (75.2%, Supplementary Table S2). The methylation signal detected with Methyl-IT was much greater than reported in the earlier PALL study^18^. A given gene may not be a DMG based on limits established in the generalized linear regression model used to identify DMGs (see Methods), but can still carry relevant DMPs. Therefore, Supplementary File S1 contains wig files with tracks for the group means of differential methylation levels between each group and the reference.

Gene-body methylation signal detected in PALL patients coincided with a significant number of genes from the list of cancer consensus genes (723) from the COSMIC database^19^: 254 DMGs, 126 DEGs, and from these, 112 DEG-DMGs.

### Network analysis on a set of differentially methylated genes (DMGs)

The preliminary application of network-based enrichment analysis (NBEA^20^) and network enrichment analysis test (NEAT^21^) on the set of DMGs permitted selection of 285 network-related DMGs (Supplementary Tables S1 and S1). Similar analysis led to selection of 326 network-related DEGs (Supplementary Table S2B, 2360 DEGs from B cells reported in Supplementary Table 3 from the original study^18^). These subsets were used to build the corresponding protein-protein interaction (PPI) networks with the STRING app of Cytoscape^16^. To bypass possible bias introduced by the heuristic used to subset the whole set of genes (NBEA^20^ and NEAT^21^), sub-clusters of hubs where retrieved by applying the MCODE Cytoscape app on the entire set of DMGs. Other than the gene list, all PPI networks analyzed in this study were built entirely based on external information retrieved from STRING database^16^.

**Figure 2:**
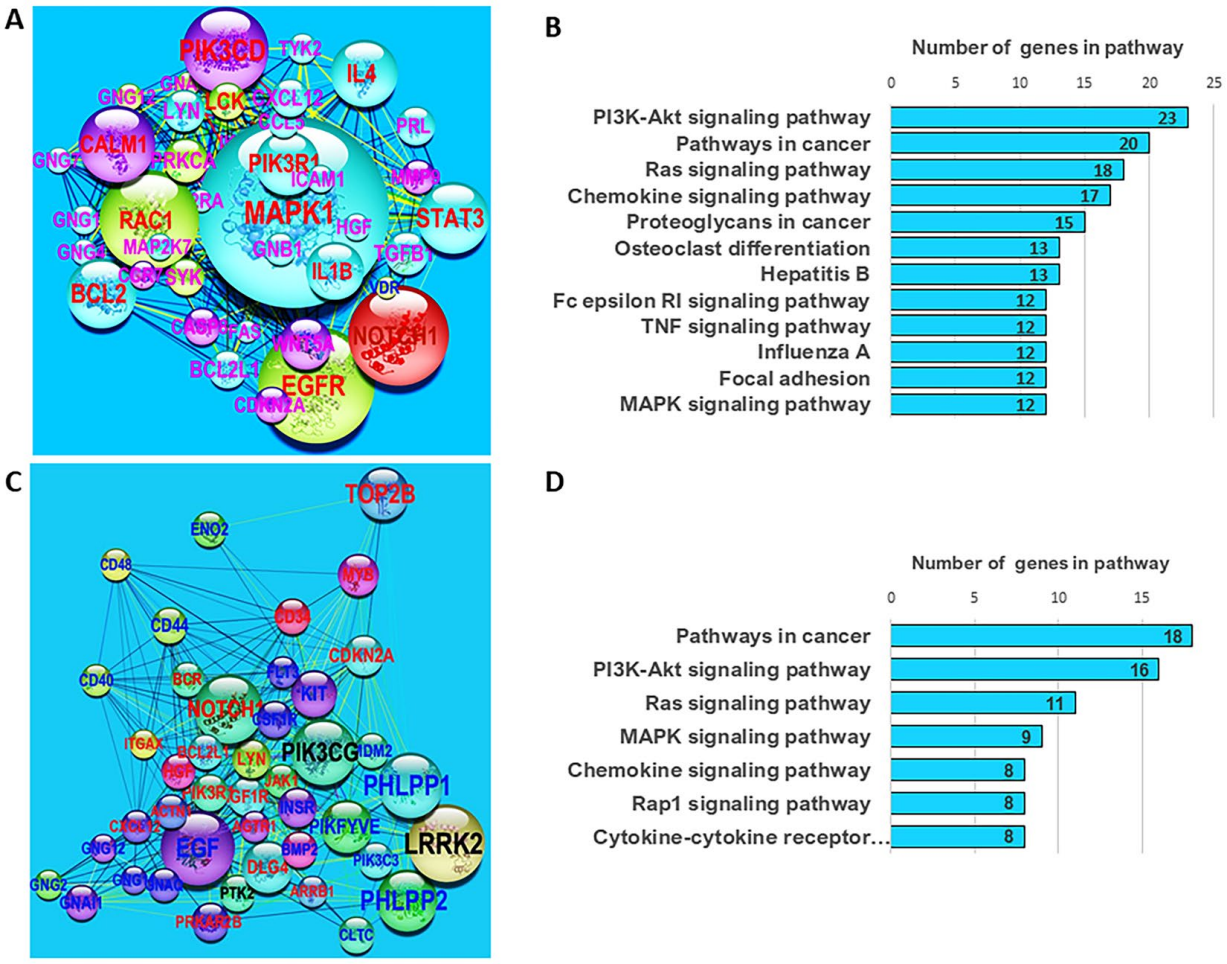
PPI subnetworks of hubs derived from subsets of network-related DMGs. (**A**) Main subnetwork of hubs obtained with the application of K-means clustering on the set of 285 network-related DMGs identified with NBEA^20^ and NEAT^21^ tests. The size of each node is proportional to its value of betweenness centrality and the label font size is proportional to its node degree. Node colors from light-green to red maps the discrete scale of logarithm base 2 of fold changes in DMP numbers for the corresponding gene: light-green: [1, 2], cyan: [2, 3], blue: [3, 4], and red: 5 or more. Edge color is based on co-expression index from white (0.042) to red (0.842). (**B**) Enrichment analysis with Cytoscape^11^ on KEGG pathway sets on network in (**A)**. (**C**) Main subnetwork of hubs obtained with the application of MCODE Cytoscape app and K-means clustering. (**D**) Enrichment analysis with Cytoscape^11^ on KEGG pathway sets on the network in **C**.

The PPI network built on the set of 285 DMGs is presented in Supplementary Fig. S2. Analysis with available tools in Cytoscape^22^ led to identification of the main hubs from the PPI network (Fig. 2A,C). Sizes of nodes and labels, as well as their colors, are used for rapid visual identification of network hubs, such that the size of each node is proportional to its value of *betweenness-centrality* and the label font size is proportional to its *node degree*^23^.

The main hub subnetworks in Fig. 2A,C were identified with the application of K-means clustering on the main networks shown in Supplementary Figs. S2 and S3, respectively, with network centralities measuring *Degree*, *Betweeness-Centrality*, *Closeness-Centrality*, *Clustering-Coefficient*, and *Average-Shortest-Path*. The bootstrap value of the mean Jaccard similarity supported the partition of DMGs into three clusters with values 0.83, 0.94, and 0.84. Generally, a valid, stable cluster should yield a mean Jaccard similarity value of 0.75 or more. Pillai statistic from MANOVA test for the three-group comparison, as well as the *F* statistic for the pairwise comparisons, were highly statistically significant, i.e., the differences between the three clusters of DMGs in terms of their network-centrality indicators were statistically significant. Network enrichment analysis of the subnetwork of hubs identified KEGG pathways involved in cancer development (Fig. 2B,D), further supporting our findings.

K-means clustering split the network of 285 DMGs (Supplementary Fig. S2) into three clusters: (*i*) the main subnetwork of hubs (46 DMGs, shown in Fig. 2A, Supplementary Table S1), (*ii*) a subnetwork with minor hubs (101 DMGs, Supplementary Fig. S4, Table S1), and (*iii*) a cluster integrated by two subnetworks (139 DMGs, Supplementary Fig. S4, Table S1). Results with MCODE Cytoscape app and K-means were consistent with those obtained by subsetting the entire set of DMGs via NBEA and NEAT^20,21^ (Supplementary Fig. S3 and Table S2), with a notable enrichment of KEGG pathways associated with cancer development (Supplementary Fig. S5).

**Figure 3:**
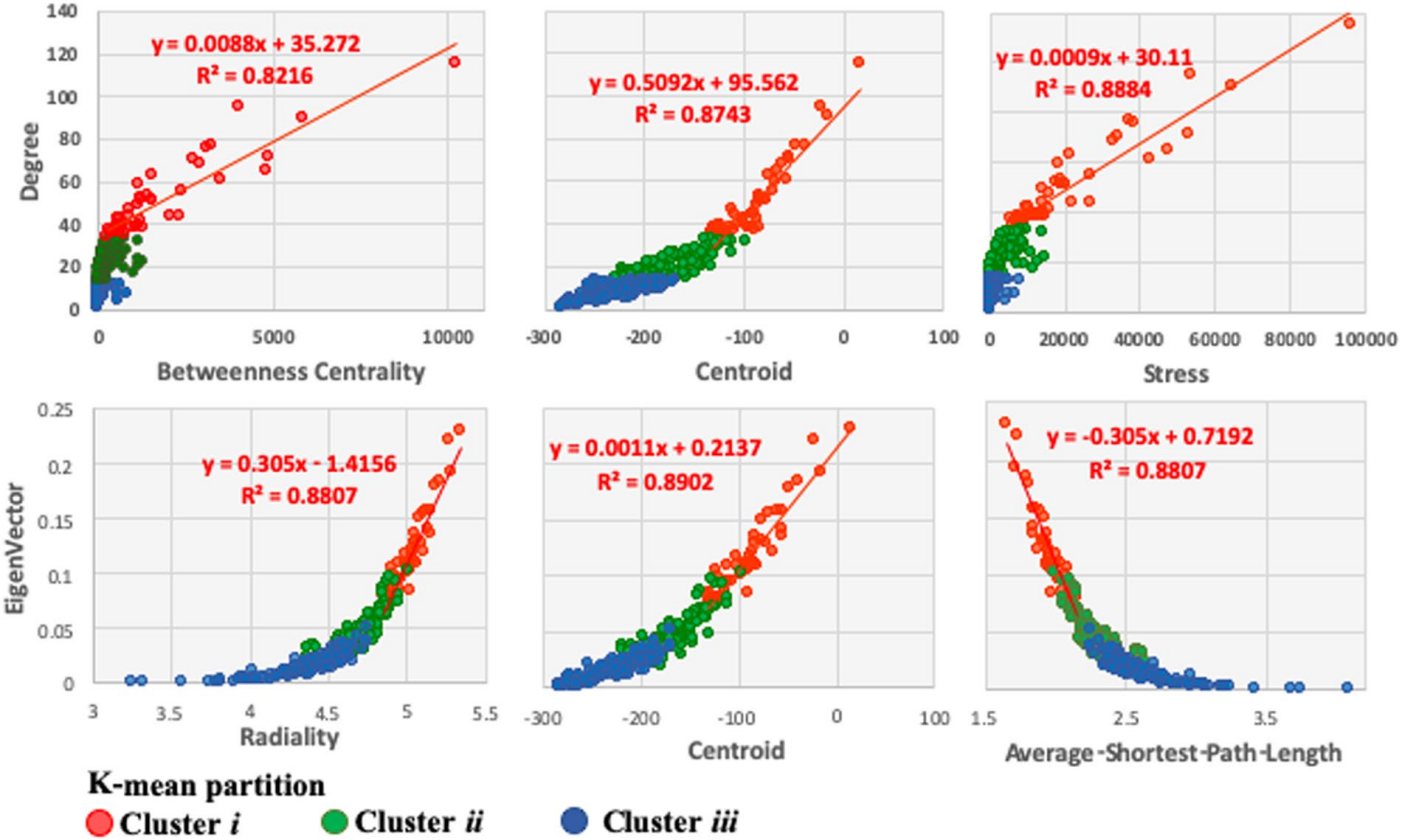
Scatter plots of network centralities measures. A general non-linear trend is notable for genes/nodes from clusters *iii* to *ii*, while the linear trend in cluster *i* can be visualized. The highest values of network centralities, *degree*, *betweenness*, *centroid*, *stress*, and *radiality*, are found in cluster *i*, which correspond to the main subnetwork of hubs presented in Fig. 2B (consistent with the lowest values of *average-shortest-path-length*). Networks from clusters *i*, *ii*, and *iii* are shown in Supplementary Fig. S4.

The scatter plots of network centrality measures (Fig. 3) suggest that the main subnetwork of hubs includes the most relevant network nodes/genes (in red) with the highest network centrality measurements. We noted a transition from a non-linear behavior, in clusters *iii* (nodes in blue) and *ii* (node in green), to a linear trend observed in cluster *i* (red points, Fig. 3). These observations suggest that the subnetwork of hubs shown in Fig. 2C also involves genes with methylation signal that have a role in PALL development^24^.

**Figure 4:**
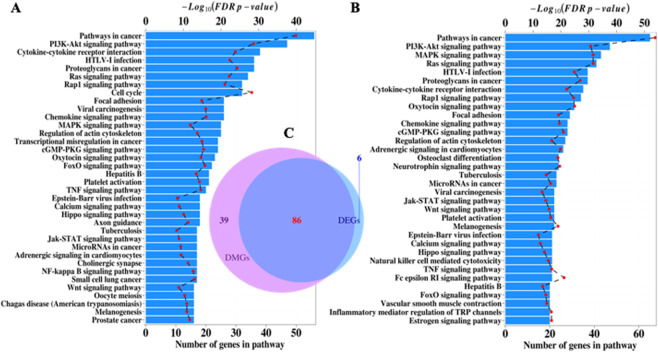
Network-based enrichment analysis of protein-protein interaction (PPI) networks independently derived from DMGs and DEGs estimated in patients with PALL. (**A**) PPI enriched network of DEGs with 15 or more genes. (**B**) PPI enriched network of DMGs with 20 or more genes. (**C**) Venn diagram with the overlapping of all PPI enriched networks of DMGs and DEGs with 7 or more genes. The PPI enriched network analysis was performed in STRING app on Cytoscape,^22,25^ and the analysis is limited to KEGG human pathways.

Results of network enrichment analysis of DMG and DEG PPI networks built with STRING (Cytoscape) are shown in Fig. 4 (Supplementary Tables S1 and S2). The analyses indicate that DMG and DEG datasets targeted many of the same pathways with overlap of 80% (Fig. 4C). Pathways linked to cancer development and apoptosis are notable, and KEGG *pathways in cancer* (hsa05200) showed pronounced enrichment, with more than 50 and 40 genes from the DMG and DEG datasets, respectively.

In the case of PALL patients, enrichment for *PI3K-Akt signaling pathway*, *MAPK signaling pathway*, *JAK-STAT signaling pathway*, *Wnt signaling pathway*, and *Focal adhesion* (all included in KEGG *pathways in cancer*) was statistically significant for both DMG and DEG subsets. The Venn diagram shown in Fig. 4C implies a high level of concordance between the enriched KEGG pathways identified in PPI networks from DEGs and from DMGs.

**Figure 5:**
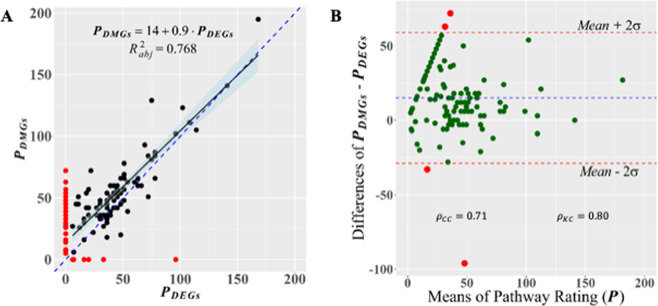
Graphical evaluation of the concordance between DEG and DMG enrichments on KEGG pathways. (**A**) scatterplot of pathway ratings (see Eq. 1) from enriched pathways on the set of DMGs $({P}_{DMGs})$ and DEGs $({P}_{DEGs})$, respectively. Regression analysis shows the linear trend of the relationship ${P}_{DEGs} > 0\,versus\,{P}_{DMGs} > 0$ (black dots). The identity dashed line (in blue) helps in gauging the degree of agreement between measurements^26^. Dots in red highlight pathways for which ${P}_{DEGs}=0$ or ${P}_{DMGs}=0$. (**B**) Bland-Altman plot of the agreement, on targeting gene pathways, between responses from gene expression and methylation regulatory systems. The agreement between measurements can also be tested by values of the Lin’s concordance correlation coefficient (${\rho }_{CC}$) and Kendall coefficient of concordance (${\rho }_{KC}$).

Figure 5 supports strong concordance between the enriched KEGG pathways identified in PPI networks from DEGs and from DMGs. Bootstrap Bayesian estimation of the Lin’s concordance correlation coefficient $$({\rho }_{cc})$$ yielded a value of $${\rho }_{cc}=0.71$$ with a confidence interval (C.I.) $$0.52\le {\rho }_{cc}\le 0.84$$, and a Kendall coefficient of concordance $${\rho }_{KC}=0.81$$ (permutation *p*-value <0.001). The linear regression analysis presented in Fig. 5A indicates a statistically significant linear relationship between the *pathway score* ($${P}_{DMG}$$) of enriched KEGG pathways in DMG PPI network (see definition at Eq. (1)) and *pathway score* ($${P}_{DEG})$$ of enriched KEGG pathways in DMG PPI network. The proximity of most of the regression points (pairs of pathways scores) around the identity line (dashed line in blue) suggests significant agreement between methylation and gene expression regulatory systems, also indicated by a regression slope of 0.9. This concordance between gene expression and methylation was graphically corroborated by a Bland-Altman plot^26^, where almost all the points are located between the *mean* − 2σ and *mean* + 2σ horizontal lines (Fig. 5B).

### DEG-DMG network analysis

**Figure 6:**
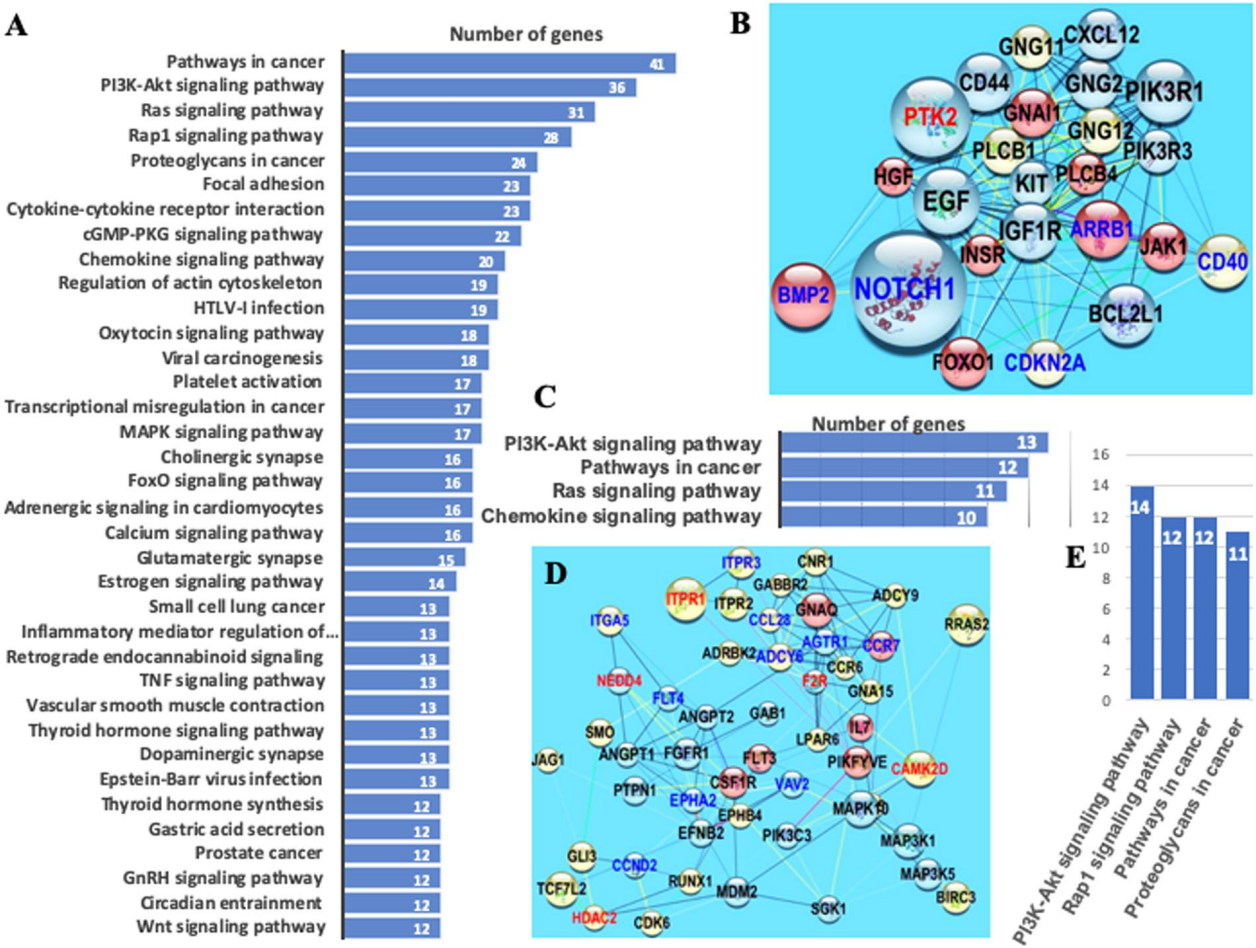
Enrichment for network-related DEG-DMGs. (**A**) Bar-plots of the enriched KEGG pathways in the PPI-network of 191 DEG-DMGs (Supplementary Fig. S6). (**B**,**D**) Subnetworks integrated by gene-hubs identified with K-means clustering of the network from panel. (**C**,**E**) Bar-plots of the enriched KEGG pathways on the networks from panels (B,D) respectively. In the networks, nodes with the same color belong to the same cluster obtained with K-Medoids clustering. To facilitate the visual identification of network hubs, node and label sizes were set proportional to the corresponding values of *betweeness centrality* and *node degree*, respectively. Edge color is based on *coexpression* index from white (0.042) to red (0.938). The PPI network and the enrichment analyses were performed in STRING app on Cytoscape^22,25^.

Three clusters were detected by applying K-means clustering on PPI-network of 191 DEG-DMGs (selection described in Methods section), and two of them integrated the subnetworks of hubs shown in Fig. 6B,D while the third cluster gave rise to several subsets of subnetworks (Supplementary Table S2). The bootstrap value of mean Jaccard similarity supported the partition of DMGs into three clusters with values 0.86, 0.96, and 0.853. Pillai statistic from MANOVA test for the three-group comparison, as well as the *F* statistic for the pairwise comparisons, were highly statistically significant, i.e., the differences between the three clusters of DEG-DMGs in their network-centrality indicators were statistically significant.

Enrichments detected in the main PPI network of 191 DEG-DMGs (Fig. 6A) and subnetworks (Fig. 6C,E. Supplementary Table S2) were consistent with previous results (Fig. 4) (*i*) focused only on the set of DMGs (not all of them DEGs, Fig. 4A) and (*ii*) focused only on the set of DEGs (not all of them DMGs, Fig. 4B).

**Figure 7:**
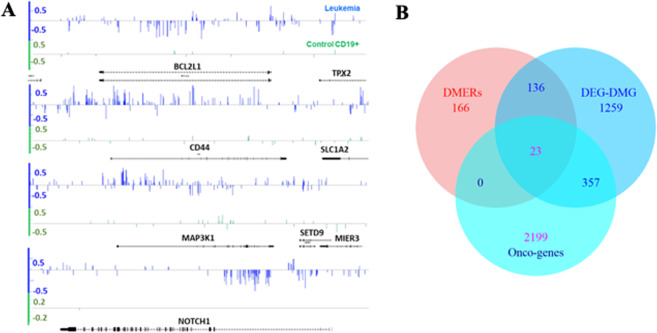
DEG-DMGs reported as cancer related gene lists. (**A**) Group mean of methylation level differences at each cytosine identified differentially methylated genes (DMGs). *BCL2L1*, *CD44*, *MAP3K1* and *NOTCH1* are linked to leukemia and other types of cancers. The genes were identified as PPI network hubs (Fig. 5B,D). Irregular distribution of methylation signal, hyper- and hypo- methylated, can be seen. Traditional DMR-based approaches fail to detect these types of variation. Methylation level differences were computed for control and treatment individuals with respect to normal CD19+ methylome from four independent blood donors used as reference. This approach provides an estimation of the natural variability of methylation changes existing in the control population. (**B**) Overlap (≥500 bp) between differentially methylated enhancer regions (DMERs) and DEG-DMGs. Although only 51 enhancers (DMERs) are activators of reported DEGs, the DMERs overlap with 159 DEG-DMG regions, of which 23 are reported oncogenes (see Methods). A total of 379 DEG-DMGs are reported oncogenes.

Group means of methylation level differences at each gene-body DMP for genes *NOTCH1*, *CD44*, and *BCL2L1* (hubs from the DMG-DEG sub-network from Fig. 6B) are shown in Fig. 7A. *NOTCH1* and *CD44* have been reported to be epigenetically regulated^27–30^ and, in particular, *NOTCH1* has been proposed as a drug target for the treatment of T-cell acute lymphoblastic leukemia^28^. *BCL2L1* is known to have roles in apoptosis and has been proposed as a drug target for cancer treatment^31^. Genes within the mitogen-activated protein kinase (MAPK) pathway are frequently altered in cancer and have been proposed as drug targets as well^32^.

Three members of this pathway are found in the DMG-DEG sub-network shown in Fig. 6D and in the DMP distribution on MAP3K1 gene-body shown in Fig. 7A. PALL-associated methylation changes were confirmed at single cytosine resolution with high classification performance^14^ (high accuracy, low false positive and false discovery rates, etc.). Methyl-IT based WGBS findings, as shown in Fig. 7A, can be further confirmed with bisulfite PCR. In total, 379 identified DEG-DMGs have been reported as cancer-related genes (Fig. 7B).

### Differentially methylated enhancer regions (DMERs)

Our initial analysis was limited to the methylation signal carried on gene-body regions. As suggested in Fig. 6, gene-associated methylation signal can also be present on genomic regions upstream and downstream to genes, including transcription enhancer regions^33^.

The methylation datasets identified 325 differentially methylated enhancer regions (DMERs). Although only 51 from the 325 identified DMERs are activators of reported DEGs (Supplementary Table S2), the list of DEG-DMG regions covered by DMERs (in at least 500 bp) totaled 159 (Fig. 7B), from which 23 were identified oncogenes.

**Figure 8:**
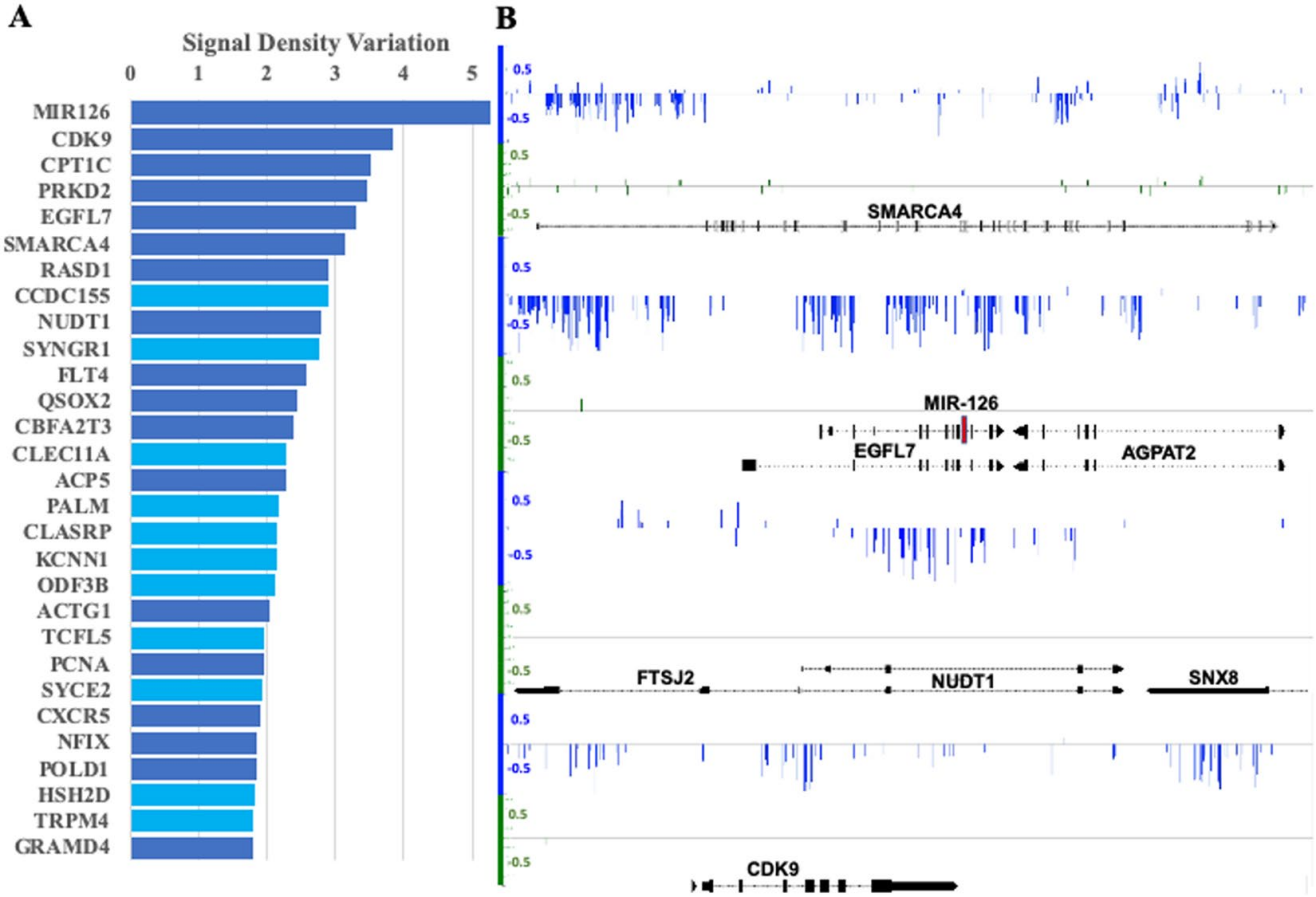
DEGs with differentially methylated enhancer regions. (**A**) Top 29 genes with highest density variation of DMP number (>1.7 DMPs/kb) in the enhancer region. Dark blue denotes genes that have been reported associated with cancer development. (**B**) Group mean of methylation level differences at each cytosine identified in differentially methylated enhancer regions corresponding to the genes *SMARCA4*, *EGRL7*, *MIR126*, *NUDT1*, and *CDK9*.

The top 29 genes with highest density variation for DMP number within enhancer regions are shown in Fig. 8. Many of these genes have been reported to be associated with cancer development and found in the sets of DMGs or DEGs. One example is the enhancer region influencing gene *EPIDERMAL GROWTH FACTOR-LIKE DOMAIN 7 (EGFL7*) and the micro-RNA *MIR-126*, both associated with cancer^34,35^. As shown in Fig. 8B, *MIR-126* resides within an intron of *EGFL7* and their enhancer region overlaps.

*MIR126* modulates vascular integrity and angiogenesis, and reports state that MIR-126 delivered via exosomes from endothelial cells promotes anti-tumor responses^36^. The hypomethylation pattern observed in the region spans a substantial part of gene *AGPAT2*, which was identified as a DMG and, although over-expressed in multiple types of cancer, was not reported as a DEG in the earlier PALL study^37^. *AGPAT2* promotes survival and etoposide resistance of cancer cells under hypoxia^38^.

### Association between methylation and gene expression

Results to date suggest the existence of an association, or at least statistical inter-dependence, between methylation and gene expression. To investigate this association, density variations of the methylation signal were quantitatively expressed by different measurements: density of methylation level difference $|\Delta {p}_{density}|$, density of total variation difference $|\Delta T{V}_{density}|$, and $|\Delta H{D}_{density}|$ (see Methods). Gene expression was shown as absolute value of the logarithm base 2 of fold change, $|lo{g}_{2}FC|$.

The association between methylation and gene expression for the current study of patients with PALL is shown in Supplementary Fig. S7. This association was corroborated by a highly significant Spearman’s rank correlation rho (*p*-value lesser than 0.001, Supplementary Fig. S7), and by two-dimensional kernel estimation (2D-KDE) and Farlie-Gumbel-Morgenstern (FGM) copula of joint probability distributions for each annotated pair of variables in the coordinate axes from the contour-plot plane (Supplementary Fig. S7).

Results indicate that methylation and gene expression show positive dependence. In general, a bivariate distribution can be considered to have a specific positive dependence property if larger values of either random variable are probabilistically associated with larger values of the other random variable^39^. According to Lai^40^, the FGM copulas shown in Supplementary Fig. S7 indicate CDM and gene expression to be *positively quadrant dependent* and *positively regression dependent*. Thus, if $X$ is the density of methylation level difference, the regression $E(Y|X=x)\,$is linear in *x*^40^. The regression of the conditional expected value of gene expression with respect to density variations of methylation signal *X* is linear in *x* (possible values of *X*). This linear trend is noticed with high joint probability in the outlined contour-plot red regions (Supplementary Fig. S7).

### PC-score of DEG-DMGs

The identification of genes playing fundamental roles in a particular phenotype is limited by the range of protein-protein interaction information in a database (STRING, in the current case). Consequently, results are mostly populated with genes from networks that are associated with diseases. To circumvent these possible biases, principal component analysis (PCA) was applied to score genes according to their discriminatory power to discern the disease state from healthy.

The first principal component (PC1) was used to build the PC-scores for DMGs, since it carried 85% of the sample variance with eigenvalues greater than 1 (Guttman-Kaiser criterion^41^, see Methods). A list of the first 11 genes with top PPI-network PC-scores is presented in Table 1, again reflecting genes associated with cancer development and supporting our interpretation that, regardless of approach, DEG-DMG datasets intersect pathways for cancer origin and progression.

**Table 1:** First 11 genes with the top PC-score based on density of methylation level differences and density of Hellinger divergences*.

Density of meth. level differences			Density of Hellinger divergence		
Gene	PC-score	Signal density variation†	Gene	PC-score	Signal density variation
COX8C	53.23	23.30	COX8C	55.10	23.30
MSC	27.02	10.50	MSC	22.14	10.50
MPEG1	16.11	8.87	MPEG1	17.36	8.87
P2RY1	15.47	5.80	BLACE	12.97	6.37
CLEC11A	15.20	6.60	CTGF	11.96	3.75
BLACE	13.20	6.37	UHRF1	11.26	5.26
UHRF1	12.08	5.26	P2RY1	11.02	5.80
EGFL7	11.95	5.64	CMTM2	9.52	3.68
ID4	11.80	5.15	CXCR5	9.34	4.63
CDK5R1	9.50	6.76	ID4	9.31	5.15
CTGF	9.13	3.75	DDIT4L	8.77	2.65

*The entire table and details are given in Supplementary Table S2. ^†^Signal density variation for each gene is given in the output of MethylIT function *countTest2*. This is the group mean difference of the normalized number of DMPs in 1k.

### Methylation signal on DEG-DMGs across individuals is network associated

Correlation and hierarchical cluster analyses of the methylation signal on genes across individuals from control and patient groups were performed to investigate the relationship of methylation signal within genes serving as network hubs and the other genes in the networks. The heat-map corresponding to the correlation matrix of methylation signal on hubs is provided in Fig. S9.

**Figure 9:**
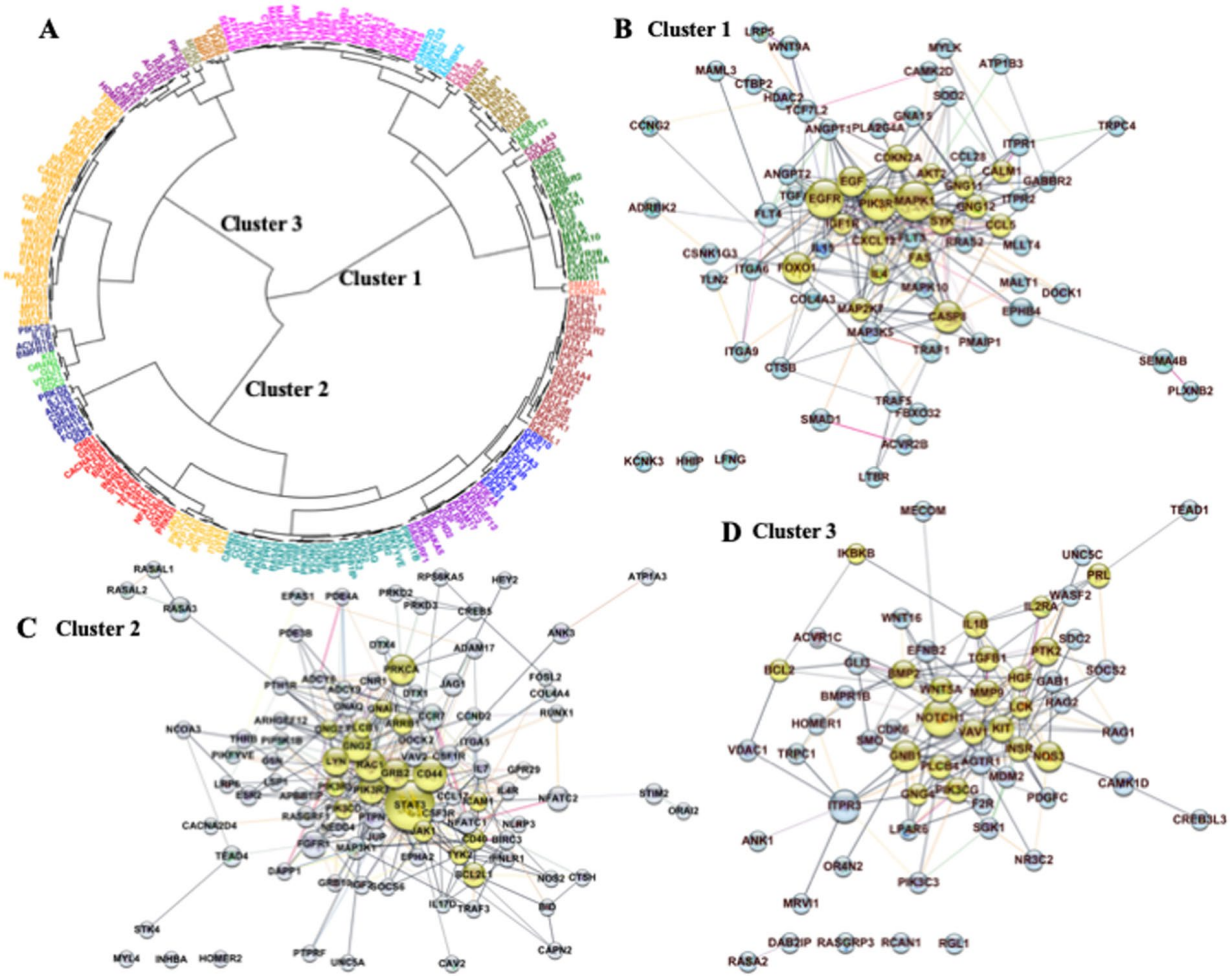
Correspondence between clusters of genes carrying correlated methylation signals and PPI networks built on them. (**A**) Hierarchical clustering on the set of 191 DEG-DMGs (Fig. 6) and DMG hubs derived from previous network analyses (see Methods). Hierarchical clustering yielded three main clusters of genes, each carrying correlated methylation signals through the individuals (Supplementary Fig. S8). (**B**–**D**) PPI networks built on the corresponding gene subsets integrating clusters 1 to 3. Not all genes from each cluster integrated the corresponding PPI network built on them; there were three genes left out of the network in (**B**) and in (**C**) and five dropped genes in (**D**). Hubs of DMGs and DEG-DMGs, identified in previous PPI network analyses (Figs. 2A,C and 6B) are highlighted in yellow. Gene co-expression is encoded in the edge colors from lighter to darker (stronger).

Hierarchical clustering on the set of DMG hubs and DEG-DMG hubs showed that their methylation signal was structured into well-defined groups (Fig. S8). This analysis was extended to genes that integrate DMG hubs and the whole DEG-DMG network (Fig. 9A). Network genes were grouped into three stable and non-arbitrary clusters. The analysis of cluster stability is in Supplementary Information S1.

Mapping of clusters into PPI networks at a medium confidence interaction score (0.4 or higher value, see Methods) is shown in Figs. 9B–D. The enrichment *p*-value for each of the three PPI networks is lesser than ${10}^{-16}$, indicating that proteins from any of clusters 1 to 3 share more interactions than would be expected for a random set of proteins of similar size drawn from the genome. Such an enrichment suggests that these sets of proteins are at least partially biologically connected as a group^16^.

Figure 9B,D shows that the hub information from each cluster structure was integrated into a central cohesive block of the corresponding mapped network (yellow nodes in Fig. 9B–D). The mapping of random gene subsets (sampled from the DMG hubs and network DEG-DMGs) into PPI networks, restricted to nodes with 3 or more interactions and confidence interaction score of 0.7, suggested that hub-core information from each cluster structure preserved in the PPI network was non-random and statistically significant. The probability of obtaining similar mappings from clusters to a cohesive block of hubs by chance is lesser than 0.05 (Supplementary Information S1).

Our results appear to support the existence of a structural association between clusters of highly correlated methylation signal on DMG hubs and DEG-DMGs and the PPI interaction networks from the STRING database^16^. Cluster integrity based on methylation signal is (mostly) preserved in the PPI networks that derive from external information on protein-protein interaction collected from published experiments in STRING. These observations insinuate that methylation repatterning is targeted.

### DMG network hubs are consistent with the centrality–lethality rule

**Figure 10:**
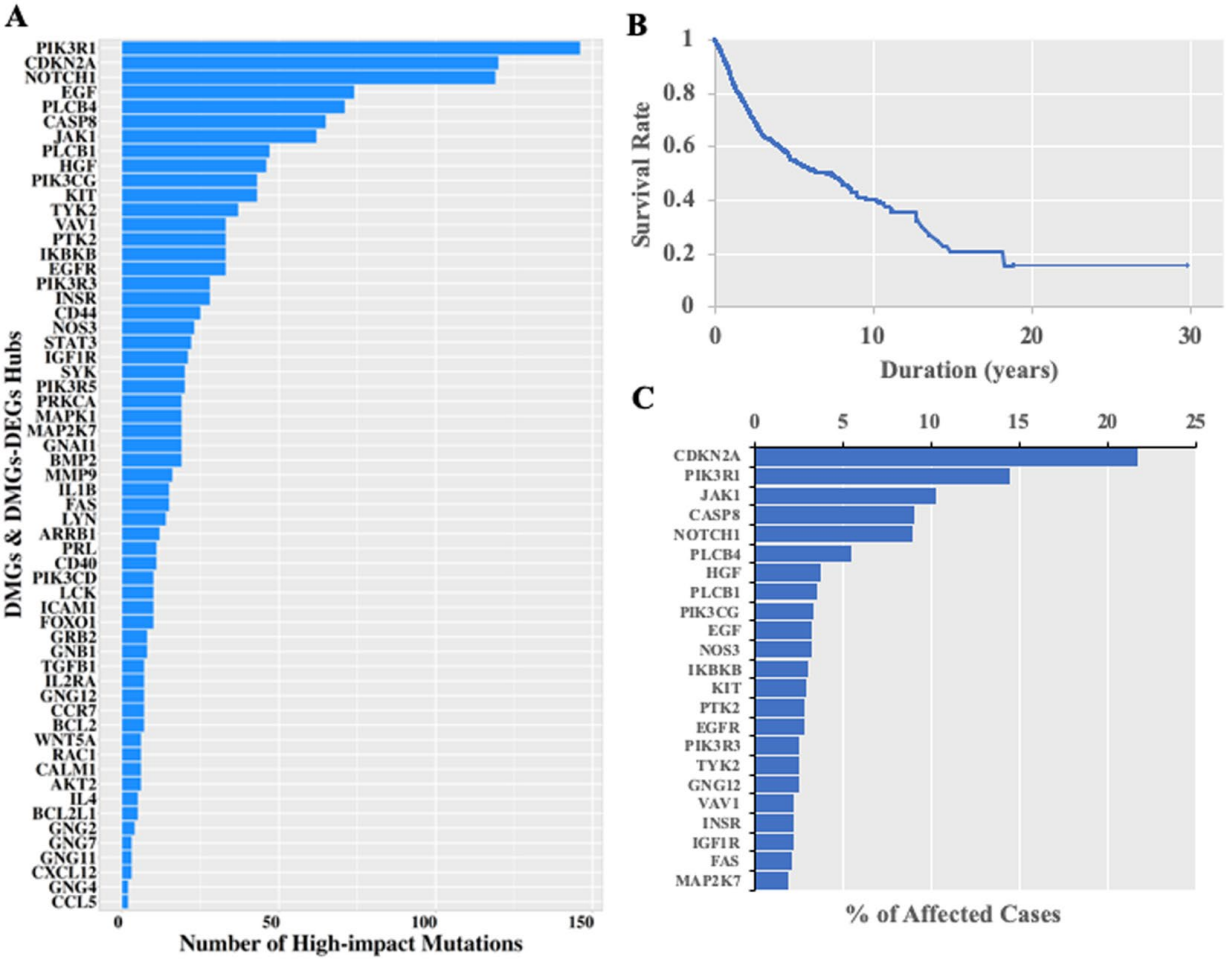
Hub essentiality expressed as impact on patient survival as reported in TCGA. (**A**) Distribution of high impact mutations across hubs. (**B**) Overall survival plot. (**C**) Distribution of most frequently mutated hubs.

The essential nature of DMG and DEG-DMG hub loci was investigated at the Genomic Data Commons Data Portal (https://portal.gdc.cancer.gov/), which contains numerous cancer datasets^42^. Screening the TCGA database revealed that all hubs identified in our analysis could undergo mutations classified as high-impact, affecting patient survival (Fig. 10).

The overlap in frequency of high-impact mutations with the hub PPI network is shown in Supplementary Fig. S10.

## Discussion

Data from this study reflect non-random methylation repatterning within gene networks reportedly associated with cancer development and risk. The data represent a novel approach to integrative network analysis of DMGs, DEGs and DEG-DMGs based on WGBS and RNA-Seq data from PALL patients.

The majority of DNA methylation changes fall within intergenic regions of the genome, and only 4795 (including non-coding) of the 57241 annotated human genes were identified as DMGs. Methylation signal is observed not only within gene-body regions of DMGs, but also (and frequently with high intensity) in upstream and downstream domains. Although we were able to identify this methylation signal with high classification performance^14^, it was not clear the extent that DMGs and DEG-DMGs may be the consequence of changes in gene expression rather than effectors. More detailed analysis of the features of methylation signal on cancer-associated genes and pathways is now feasible among the identified sites.

Since networks were built based entirely on external information retrieved from STRING database, their analysis provided unbiased identification of network hubs independent of our information on methylation or gene expression. Network enrichment analysis of DMGs identified several KEGG pathways of relevance to cancer. Relevant genes were identified as network hubs and grouped into clusters of network hubs carrying highest network centrality measurements (Figs. 2 and 6). Available theoretical and experimental evidence suggests that disruption of a network hub, or genes that regulate the hub, could alter the entire gene network^6,7,24,43^. Genomic studies indicate that mutations of a highly connected protein node (hub) are more likely to be lethal to an organism than mutations at a sparsely connected node (non-hub), a phenomenon known as the centrality-lethality rule. Thus, hub identification may offer candidate targets in the search for potential biomarkers. Strong linearity trends observed in pairwise regression between the centrality measurements (Fig. 3) in the main hub cluster (Fig. 2A) suggest that genes from the cluster are non-randomly targeted by methylation action during PALL development^24^.

Clusters of hubs integrating PPI subnetworks comprise the backbone of a network. The essentiality of gene hubs in preserving the integrity of the interacting network is quantitatively expressed in network centrality statistics. For sub-networks of hubs (Figs. 2 and 6), higher centrality values and linear relationships between the centrality statistics of the network hubs reflects a higher number of reported biologically meaningful associations between the hubs and the other genes in the sub-networks and main network (Fig. 3).

There was strong correspondence in the network enrichment analyses derived from PPI networks in DMGs and DEGs (Fig. 4), supporting the non-random nature of methylation signals within protein-coding regions in signaling pathways linked to cancer development. Although not all DEGs are detected as DMGs and vice versa, massive overlap of enriched KEGG pathways (Fig. 4) suggests a coordinated response of methylation and gene-expression machineries. The possibility of such an *in concert* regulatory response was statistically supported by Lin’s concordance correlation coefficient and Kendall coefficient of concordance.

An example of coordinated regulatory response of methylation and gene expression is seen in the case of the *EGFR* gene, identified as a hub in the DMG network (Fig. 2). *EFGR* is a tyrosine kinase that regulates autophagy via the PI3K/AKT1/mTOR, RAS/MAPK1/3 (enriched pathways shown in Fig. 4A,B, and in Fig. 6A,E), and STAT3 signaling pathways^44,45^. Although EGFR was not a reported DEG, its activators, *EPIDERMAL GROWTH FACTOR* (*EGF*, Fig. 6B) and *EGFL7* were identified as both DMGs and DEGs. *EGFL7* is reported to be a key factor for the regulation of the *EGFR* signaling pathway^46^. Additionally, *EGFL7* is a secreted angiogenic factor that can result in pathologic angiogenesis and enhance tumor migration and invasion via the *NOTCH* signaling pathway^34^ (a pathway enriched in the PPI-DMG network). The *NOTCH* pathway is a conserved intercellular signaling pathway that regulates interactions between physically adjacent cells. In the set of patients with PALL, *NOTCH1* is reported as a DEG and DMG (Figs. 2A and 6B).

Another example of the gene network architecture of leukemia emerges by tracking up- and downstream interconnections of the genes *PIK3CG* (DEG-DMG) and *PIK3CD* (a DMG network hub, Fig. 2) from the *PI3K/AKT* signaling pathway (enriched in the set of DEG-DMGs, Fig. 6). Phosphatidylinositol-4,5-bisphosphate 3-kinase (*PI3K*) is a critical node in the B cell receptor (BCR, a DEG-DMG) signaling pathway and its isoforms, *PIK3CD* and *PIK3CG* are involved in B cell malignancy^47^. Crosslinking CD19 with the BCR augments PI3K activation, and VAV proteins, VAV1 (DMG), VAV2 (DEG-DMG), and VAV (DEG-DMG) also contribute to PI3K activation downstream of BCR and related receptors^48^. BCR and its downstream signaling pathways, including Ras/Raf/MAPK, JAK/STAT3, and PI3K/AKT (all enriched in PALL patients, Figs. 4 and 6), play important roles in malignant transformation of leukemia^49^.

Our analysis also considered gene regulatory domains upstream and downstream to gene-body regions and, in particular, enhancer regions. The set of genes targeted by DMERs did not integrate to a PPI network, but were found in signaling pathways or regulators from them. As in the previous analyses, enhancer methylation repatterning identified cancer-related genes (Fig. 7B). For example, *SMARCA4* (Fig. 8) encodes an ATPase of the chromatin remodeling SWI/SNF complexes frequently found upregulated in tumors^50^ and represents a DEG-DMG in patients with PALL. The product of this gene can bind BRCA1 (DEG-DMG)^51^ and also regulates the expression of the tumorigenic protein CD44 (DEG-DMG)^52^.

PPI networks are only models to identify highly interconnected players from the subjacent web architecture of genes involved in a specific biological process. Thus, results from the application of more than one network model can complement, and different network models do not necessarily overlap 100% with the set of enriched pathways. Deriving subsets of the DEG-DMG dataset by applying MCODE clustering increased confidence over previous results.

The integrative analyses of DMGs, DEGs and the networks derived from them, as well as DMERs (graphically summarized in Figs. 2 to 10), provided consistent indication related interactions with cancer development and an association between gene methylation repatterning and gene expression changes. This association was supported by Spearman’s rank correlation rho and the bivariate FGM copula (Supplementary Fig. S7), which implies a linear dependence for expected values of gene expression changes on methylation changes for the set of DEG-DMGs.

Our analysis suggested a *stochastic deterministic dependence* relationship, where larger values of gene expression changes are probabilistically associated with larger values of methylation changes (in the entire set of 1772 DEG-DMGs). Within the set of DEG-DMGs, observed changes in gene expression were not statistically independent of the methylation changes, showing association with a significant linear trend (Supplementary Fig. S7). This result may be indication that the relationship between gene methylation repatterning and altered gene expression would be present at lower density methylation levels. This relationship could be overlooked with over-stringent filtering of methylome data. Three analytical approaches assisted in discovering this association: *i*) signal detection for DMP identification, *ii*) GLM-based group comparison for DMG identification, and *iii*) copula modeling of stochastic dependence.

Results demonstrated the potential of integrative network analysis of DMGs and DEGs for identification of biologically relevant methylation biomarkers. Numerous clusters of interacting genes were detected in the sub-networks of hubs from PPI networks of DMGs and DEGs, with only a few described here. Hubs of DMGs and DEG-DMGs sharing similarity in their methylation patterns across patients (Fig. 9A) were located in cohesive blocks in the PPI network (Fig. 9B,D). This observation suggests, with support of statistical analysis, that these hubs were not arbitrarily targeted with methylation changes, and may be consistent with susceptibility of the hubs to high impact mutations (Fig. 10).

More detailed analysis of these data leads us to propose three factors likely to be important to biomarker identification. A potential biomarker must 1) be a DMG or a DEG-DMG with one or more well defined differential methylation pattern(s) on gene-body, upstream or downstream domains; 2) integrate one or more gene pathways that are biologically relevant for leukemia and, simultaneously, show enrichment in the PPI networks of DMGs and DEGs, and 3) represent a hub or a biological connection to a relevant hub. Genes holding to these guidelines integrate the subnetworks of hubs shown in Figs. 2B, 5C,D and 9, and the list of potential biomarkers can be extended using the information provided in the Supplementary Tables S1 and S2.

As shown in Figs. 7 and 8, potential biomarkers need not comprise entire genes, but more likely specific regions within or neighboring gene regions. The last intron in *NOTCH1* (Fig. 7) or the region covering *MIR-126* in *EGFL7* (Fig. 8) are candidate examples. Intersection of the identified networks with available data from independent cancer studies lends support for this approach in identifying effective disease biomarkers. However, while intersection of methylome and gene expression data with cancer-relevant gene networks is compelling, we have not eliminated the possibility that these outcomes may be influenced by the relative abundance of cancer-related networks within the various databases currently available. To help circumvent this limitation, we proposed ranking the DEG-DMGs based on their discriminatory power to discern disease state from healthy.

Potential biomarkers can be scored with the application of PCA (Table 1 and Supplementary Table S2). In this study, the first PC was sufficient to build a PC-score of DEG-DMGs based on gene-body methylation signal intensity. PC-scores identify cancer-related genes that are not identified by the PPI network approach, although not all relevant genes were identifiable, including, for example, *NOTCH1*. Within a large gene like *NOTCH1*, the non-homogenous distribution of gene body methylation signal (Fig. 7A) can result in what appears as low-density methylation signal globally, even with high local signal. Nevertheless, PC-score provides an acceptable complement to the PPI network approach. Results obtained with the approach proposed here support its application for identification of reliable and stable biomarkers. A list of genes relevant as biomarker candidates for leukemia, several previously proposed as biomarkers by others, is provided in the Supplementary Tables online.

## Materials and Methods

### Methylation and gene expression datasets

The datasets of genome-wide methylated and unmethylated read counts (for each cytosine site) from normal CD19+ blood cell donor (NB) and from patients with pediatric acute lymphoblastic leukemia (PALL) where downloaded from the Gene Expression Omnibus (GEO) database. DMPs were estimated for control (NB, GEO accession: GSM1978783 to GSM1978786) and for patients (ALL cells, GEO accession number GSM1978759 to GSM1978761) relative to a reference group of four independent normal CD19+ blood cell donor (GEO accession: GSM1978787 to GSM1978790). The datasets of DEGs from the group of patients with PALL were taken from the Supplementary Information provided in the previous study^18^.

A list of 2,579cancer-related genes compiled by Bushman Lab (http://www.bushmanlab.org/links/genelists) was used to identify DEG-DMGs oncogenes.

### Methylation analysis

Methylation analysis was performed by using our home pipeline Methyl-IT^14^ version 0.3.1 (a R package available at https://git.psu.edu/genomath/MethylIT). Estimation of differentially methylated positions (DMPs) is consistent with the classical approach using Fisher’s exact test except for a further application of signal detection (see examples of methylation analysis with MethylIT at https://github.com/genomaths/MethylIT, version 0.3.2). Need for the application of signal detection in cancer research was pointed out decades ago^53^. Here, application of signal detection was performed according to standard practice in current implementations of clinical diagnostic tests^54–56^. That is, optimal cutoff values of the methylation signal were estimated on the receiver operating characteristic curves (ROCs) based on ‘Youden Index’^54^ and applied to identify DMPs. The decision of whether a DMP is detected by Fisher’s exact test (or any other statistical test implemented in Methyl-IT) is based on optimal cutoff value^55^.

Differentially methylated positions (DMPs) were estimated for control (four normal CD19+ blood cell donors) and patient (ALL cells from three patients) groups relative to a reference group of four independent normal CD19+ blood cell donors. Inclusion of a reference group permitted the evaluation of natural variation in healthy individuals and reduction of noise in a signal detection step of the methylation analysis pipeline.

#### Estimation of differentially methylated regions (DMRs)

The regression analysis of the generalized linear model (GLMs) with logarithmic link, implemented in MethylIT function *countTest*, was applied to test the difference between groups of DMP numbers/counts at specified genomic regions, regardless of direction of methylation change. Here, the concept of DMR is generalized and it is not limited to any particular genomic region found with specific clustering algorithm. It can be applied to any naturally or algorithmically defined genomic region. For example, an exon region identified statistically to be differentially methylated by using GML is a DMR. In particular, a DMR spanning a whole gene-body region shall be called a DMG. DMGs were estimated from group comparisons for the number of DMPs on gene-body regions between control (CD19+ blood cell donor) and ALL cells based on generalized linear regression.

The fitting algorithmic approaches provided by *glm* and *glm.nb* functions from the R packages *stat* and MASS were used for Poisson (PR), Quasi-Poisson (QPR) and Negative Binomial (NBR) linear regression analyses, respectively. These algorithms are implemented in the Methyl-IT^14^ function *countTest* and *countTest2*, which only differ in the way to estimate the weights used in the GLM with NBR. The following *countTest* parameters were used: minimum DMP count per individual (8 DMPs), test *p*-value from a likelihood ratio test (test = “LRT”) and *p*-value adjustment method (Benjamini & Hochberg^57^), cut off for *p*-value (α = 0.05), and *Log2Fold* Change for group DMP number mean difference >1.

The methylation analysis of genomic regions to identify differentially methylated enhancer regions (DMERs) was performed on a set of enhancers reported by Hnisz *et al*.^58^. Usually, the size of the genomic region covered by an enhancer varies depending on the tissue type. In our current case, for each enhancer we analyzed the maximum region spanning all reported sizes for different tissues.

### Network analysis

Protein-protein interaction (PPI) networks were built with the STRING app of Cytoscape^16,22^. Network analyses were conducted in Cytoscape. When applying network analysis, not all DMGs and DEGs estimated from the experimental datasets integrate networks. Working with subsets of the most relevant genes from the experimental dataset able to integrate networks helped facilitate further network analysis. When the number of genes exceeded l00 for network analysis, biologically meaningful web connections were difficult to visualize. Therefore, subsetting was applied to select network-related genes.

Biologically relevant subsets of network related genes were selected from the entire set of genes (DMGs, DEG, or DEG-DMGs) by using the R packages NBEA and NEAT^20,21^. Alternatively, Cytoscape app MCODE was used for subsetting an entire network^59^. PPI subnetworks from four network modules identified with MCODE are shown. MCODE parameters for degree cutoff: 10, node density cutoff: 0.01, node score cutoff: 0.2, K- score 10, and max. depth: 100. K-mean clustering algorithm was applied to each subnetwork to obtain subnetworks of hubs using the following node attributes for clustering: *betweenness-centrality*, *degree, closeness-centrality*, and *clustering coefficient*. Cluster bootstrapping was applied to evaluate the stability of the cluster found with K-means based on Jaccard similarity. The computation was performed with the function *clusterboot* from R package *fpc* (version 2.2.3).

To facilitate the visual identification of network hubs, node and label sizes were set based on the node *betweenness-centrality* and *degree* measures, where size of each node (in PPI network) was proportional to its value of *betweenness-centrality* and label font size was proportional to its *node degree*^23^. Network enrichment analysis in KEGG pathways followed each graphic subnetwork.

To build the hierarchical clustering presented in Fig. 9, the Pearson correlation coefficient of methylation signal on genes (through individuals) was transformed to a dissimilarity measure: 1 – *corr*(*x*, *y*), where *corr*(*x*, *y*) stands for the correlation between genes *x* and *y*. The heatmap corresponding to the dissimilarity matrix is shown in Supplementary Fig. S8. Ward’s minimum variance method was used as agglomeration algorithm. The methylation signal on each gene was expressed as density of Hellinger divergences of methylation levels at each DMP in the gene region. Methylation signals were computed using function *getGRegionsStat* from the R package MethylIT.utils (version 0.1), available at https://github.com/genomaths/MethylIT.utils.”

To evaluate whether the methylation signal was associated to a PPI network, clustered genes were mapped into STRING PPI networks (Fig. 9). The uncertainty in hierarchical clusters from Fig. 9 was evaluated with the R package *pvclust* (version 2.0). For each cluster in hierarchical clustering, *p*-values were estimated via multiscale bootstrap resampling. Results are given in Supplementary Information S1.

For each cluster, the amount of information preserved in the mapping was estimated by the fraction of genes from the given cluster that integrated a main network with at least a minimum required interaction score. The confidence scores indicate the estimated likelihood that a given interaction is biologically meaningful, specific and reproducible, given the supporting evidence.

### Concordance test for DEG and DMG enrichments on KEGG pathways

The concordance between DEG and DMG enrichments on KEGG pathways, derived from the PPI network via STRING app in Cytoscape, was evaluated with the application of the Lin’s concordance correlation coefficient (${\rho }_{cc}$) and Kendall coefficient of concordance (${\rho }_{KC}$). The R package *agRee* was used for the bootstrap Bayesian estimation of ${\rho }_{CC}$ point value and confidence interval^60^; while the R package *vegan* was used to compute ${\rho }_{KC}$ through a permutation test^61^.

To perform the concordance test, a score was assigned to each enriched KEGG pathway from DEGs and DMGs based on the *number of genes in the pathway* and on its corresponding *statistical signification* based on its FDR *p*-value. Only pathways with FDR *p*-value lesser than 0.0004 were considered. A new variable, statistical signification (*sig*) was defined according with the scale: $sig=$ 1, 2, 3, for *p*-values in the intervals (${10}^{-5},\,{10}^{-4}\,$), (${10}^{-6},{10}^{-5}\,$), and ($0,{10}^{-6}\,$), respectively. The valor of $sig=0$ was assigned to pathways not enriched in one of the group, DEGs or DMGs. For example, *Phosphatidylinositol signaling system* was not enriched in the set of PPI-DMGs and, consequently $si{g}_{DMG}=0$, but it was enriched in the set of PPI-DEGs with $si{g}_{DMG}=3$. Then, a new variable, named *pathway score* was defined according to the formula:

1 $$P=\#\,of\,genes\,in\,pathway\,\times sig$$

We would use the notation ${P}_{k}^{i}$ to indicate that the rating was performed for pathway $i$ identified on the gene set *k* (*k =DMGs, DEGs*). That is, the pathway score *P* not only carries information on how many genes are found on each pathway but also information on the enrichment statistical signification. The estimated values of ${P}_{DMG}^{i}$ and ${P}_{DEG}^{i}$ for each enriched pathway $i$ (from DEGs and DMGs sets, respectively) were used in the concordance tests and in the Bland-Altman plot (Fig. 5B).

### Stochastic association between methylation and gene expression

Methylation density of gene regions simultaneously identified as DEGs and DMGs was expressed in terms of different magnitudes: 1) ${p}_{density}^{i}$, density of methylation levels (*i*: control or patients); 2) $T{V}_{density}^{i}$, density of the difference of methylation levels between each group (control or patients) and an independent group of four healthy individuals (reference group); 3) $TV{B}_{density}^{i}$, $TV$ with Bayesian correction, and 4) $H{D}_{density}^{i}$, density of Hellinger divergence, where *i* denotes the group mean, control or patient. The density in 1000 bp of a variable *X* at a given gene region was defined as the sum of the magnitude *X* divided by the length of the region and multiplied by 1000. The differences in methylation densities between control and patient groups were estimated as the absolute difference of methylation levels $|{X}_{density}^{control}|=|{X}_{density}^{control}-{X}_{density}^{patient}|$, where ${X}_{density}^{i}$ represents one of the mentioned variables. Methyl-IT R package provides all the functions to obtain all variables mentioned here (https://github.com/genomaths/MethylIT (version 0.3.2) and https://github.com/genomaths/MethylIT.utils).

Spearman’s rank correlation $\rho$ (rho) was estimated to evaluate the association between the pairs of variable $|\Delta lo{g}_{2}FC|$ versus:$\,|\Delta {p}_{density}|$, $|\Delta T{V}_{density}|$, $|\Delta TV{D}_{density}|$, and $|\Delta H{D}_{density}|$. Since correlation analysis only measures the degree of dependence (mainly linear) but does not clearly discover the structure of dependence, we further investigate the structural dependence between these variables with application of Farlie-Gumbel-Morgenstern (FGM) copula. FGM copula model estimation was performed with R package copula^62^.

### Principal component analysis (PCA)

PCA is a standard statistical procedure to reduce data dimensionality, to represent the set of DMGs by new orthogonal (uncorrelated) variables, the principal components (PCs)^63^, and to identify the variables with the main contribution to the PCs carrying most the sample variance. A PC-based score (PC-score) was built by ranking the DEG-DMGs based on discriminatory power to discern between the disease state and healthy. Each individual was represented as vector of the 1775-dimensional space of DEG-DMGs. Two PC-scores were estimated: the first based on the density of Hellinger divergence on the gene-body and the second one based on the density of the absolute value of methylation level difference. The density of a magnitude *x* is defined as the sum of *x* at each DMP divided by the gene width (in base-pairs). The first principal component (PC1) was used to build a PC-based score for the DEG-DMG set, since it had an eigenvalues (variance) greater than 1 and carried more than 85% of the whole sample variance (Guttman-Kaiser criterion^41^). The PC-score was built using the absolute values of the coefficients (loadings) in PC1 for each variable (gene). Since the sum of the squared of variable loadings over a principal component is equal to 1, the squared loadings tell us the proportion of variance of one variable explained by the given principal component. Thus, the greater is the PC-score value, the greater will be the discriminatory power carried by the gene.

The density of *HD* on the gene-body was computed with MethylIT function *getGRegionsStat* and the principal component with function *pcaLDA*, which conveniently applies the PCA calling function *prcomp* from the R package ‘*stats*’.

## Supplementary information

Supplementary Figures and Information.

Dataset 1.

Dataset 2.

Supplementary File S1

## Data Availability

All the methylome datasets and software used in this work are publicly available. The Methyl-IT R package used in the DMP and DMG estimations, as well as several examples on how to use Methyl-IT, are available at GitHub: https://github.com/genomaths/MethylIT (version 0.3.2). The datasets supporting conclusions of this report are included within Supplementary Material. R script evaluating Methyl-IT performance are available at https://git.psu.edu/genomath/MethylIT_examples. Methyl-IT version 0.3.1, used to compute DMGs, is available at https://git.psu.edu/genomath/MethylIT. For new analyses we recommend using the current version 0.3.2 available at https://github.com/genomaths/MethylIT.
