# Supplementary material for: Integrative Network Analysis of Differentially Methylated and Expressed Genes for Biomarker Identification in Leukemia
Source: Sci Rep. 2020 Feb 7;10:2123. doi: 10.1038/s41598-020-58123-2 (PMC7005804; doi:10.1038/s41598-020-58123-2)

Supplementary Figures

Manuscript title:
Integrative Network Analysis of Differentially Methylated and Expressed Genes for Biomarker Identification in Leukemia

Robersy Sanchez and Sally A. Mackenzie

Supplementary Figure S1. Distribution of methylation changes on chromosome and gene-body. **A**, Distribution of methylation changes at DMP positions on selected chromosomes as viewed within genome browser. **B**, boxplot of the means of methylation levels on chromosomes and at genes. In all cases, patient (P) data are in blue and control (C) in green.

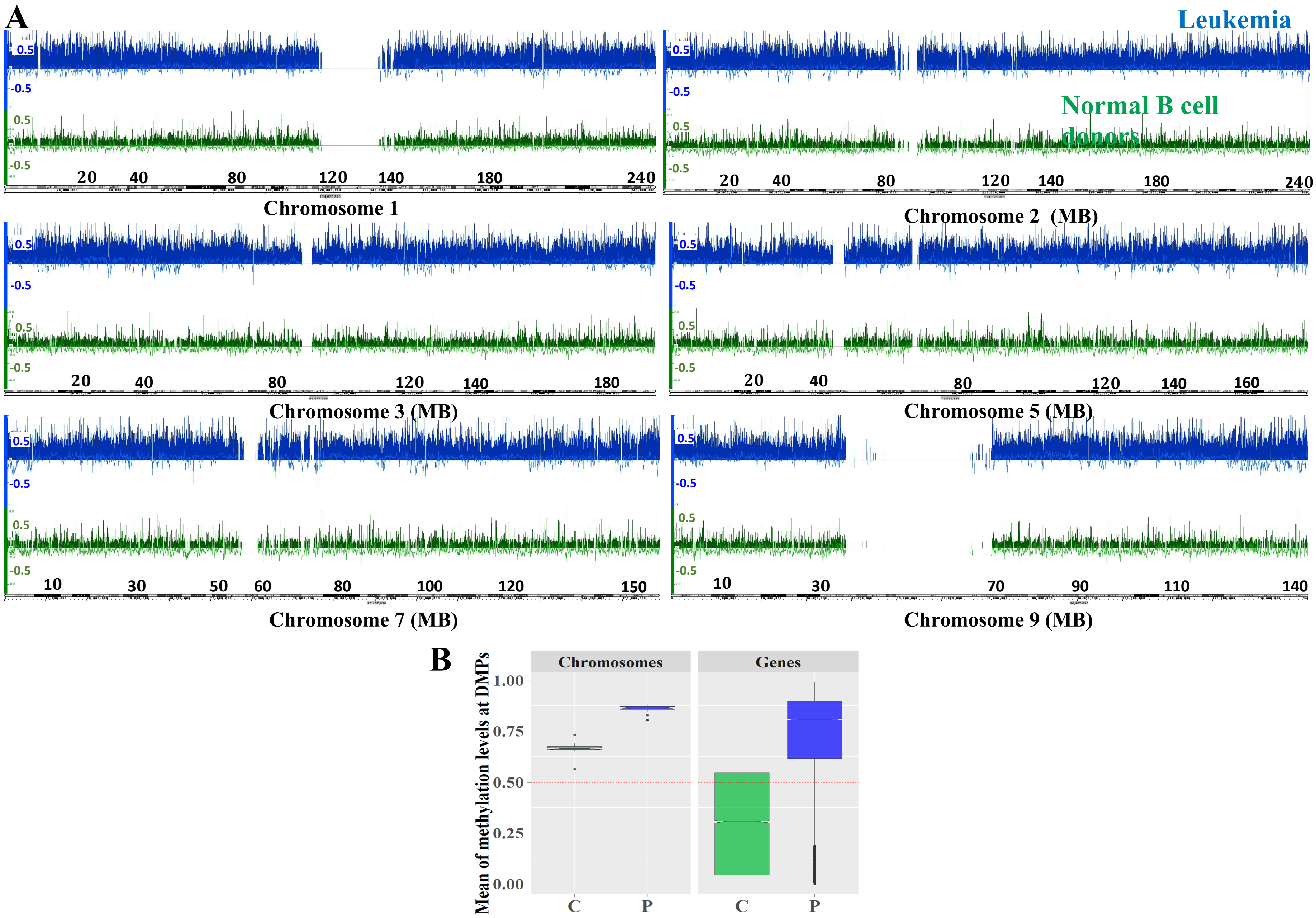
A
Leukemia
Normal B cell donors
0.5
-0.5
Chromosome 1
Chromosome 2 (MB)
Chromosome 3 (MB)
Chromosome 5 (MB)
Chromosome 7 (MB)
Chromosome 9 (MB)
B
Chromosomes
Genes
Mean of methylation levels at DMPs
1.00
0.75
0.50
0.25
0.00
C
P

Supplementary Figure S2. PPI networks built on the subset of 285 network-related DMGs. The size of each node is proportional to its value of betweeness centrality and the label font size is proportional to its node degree. Node colors from light-green to red maps the discrete scale of logarithm base 2 of fold change in DMP number for the corresponding gene: light-green: [1, 2), cyan: [2, 3), blue: [3, 4), and red: 5 or more. **B**, a subnetwork with minor hubs (101 DMGs). **C**, a cluster (139 DMGs) integrated by two subnetworks.

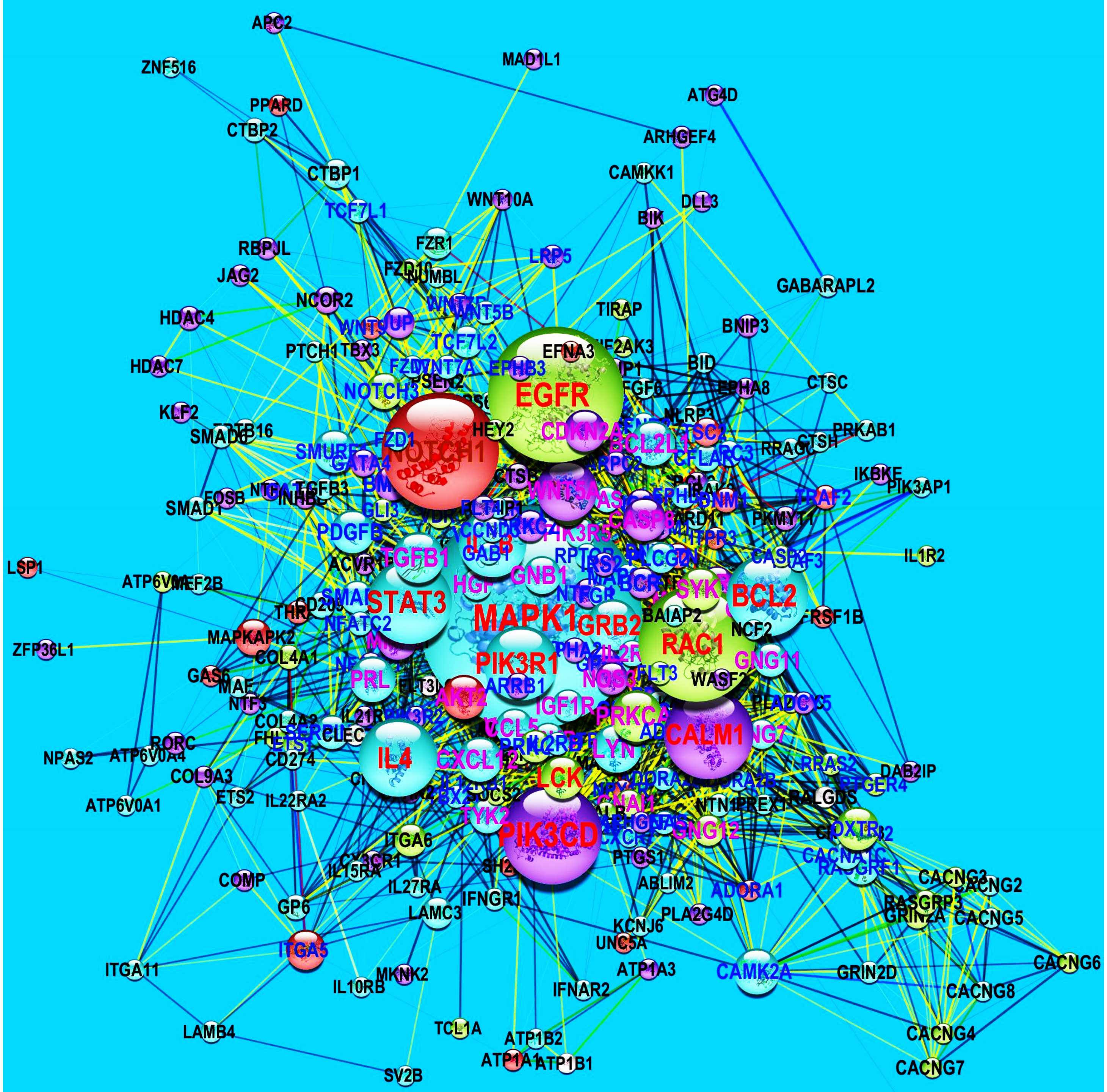

APC2
ZNF516
MAD1L1
PPARD
ATG4D
CTBP2
ARHGEF4
CTBP1
CAMKK1
WNT10A
DLL3
TCF7L1
BIK
FZR1
LRP5
RBPJL
FZD10
NUMBL
JAG2
GABARAPL2
NCOR2
TIRAP
HDAC4
WNT5B
BNIP3
PTCH1
TBX3
TCF7L2
HDAC7
EFNA3
BID
EPHB3
PSEN2
EGFR
FGF6
EPHA8
CTSC
NOTCH3
KLF2
HEY2
NLRP3
PRKAB1
SMAD6
CDKN2A
BCL2L1
SMURF1
FZD1
NOTCH1
CTSH
GATA4
CFLAR
IKBKE
TGFB3
CTSB
PIK3AP1
FOSB
WNT5A
TRAF2
SMAD1
INHBE
GLI3
NRD1
PDGFB
PIK3R5
CASP8
GAB1
RPTOR
IRS2
IL1R2
LSP1
TGFB1
GNB1
CASP2
ATP6V0A2
MEF2B
HGF
BCL2
STAT3
MAPK1
GRB2
BAIAP2
NFATC2
RAC1
NCF2
MAPKAPK2
ZFP36L1
COL4A1
PIK3R1
GNG11
GAS6
PRL
ARRB1
FLT3
WASF2
MAF
AKT2
NTF3
IGF1R
PRKCA
COL4A2
CCL5
CALM1
RORC
IL4
CXCL12
LYN
NPAS2
ATP6V0A4
CD274
RRAS2
DAB2IP
LCK
COL9A3
ETS2
IL22RA2
PTGER4
ATP6V0A1
NTN1
RALGDS
SOCS2
TYK2
PIK3CD
GNG12
OXTR
ITGA6
PTGS1
CACNA1C
CX3CR1
IL15RA
COMP
ABLIM2
CACNG3
CACNG2
IL27RA
ADORA1
IFNGR1
GP6
LAMC3
RASGRP3
GRIN2A
PLA2G4D
CACNG5
KCNJ6
UNC5A
ITGA5
CACNG6
ITGA11
ATP1A3
CAMK2A
GRIN2D
MKNK2
IL10RB
IFNAR2
CACNG8
LAMB4
TCL1A
CACNG4
ATP1B2
ATP1A1
ATP1B1
SV2B
CACNG7

Supplementary Figure S3. PPI subnetwork module derived with Cytoscape app MCODE from the PPI network of 1775 DEG-DMGs. Node colors from yellow to red maps the discrete scale of logarithm base 2 of fold changes in gene expression for the corresponding gene: yellow: lesser or equal to -6, …, light-green: (-2, -1], …, cyan: (2, 3], … blue: (4, 5], …, red: 10 or more.

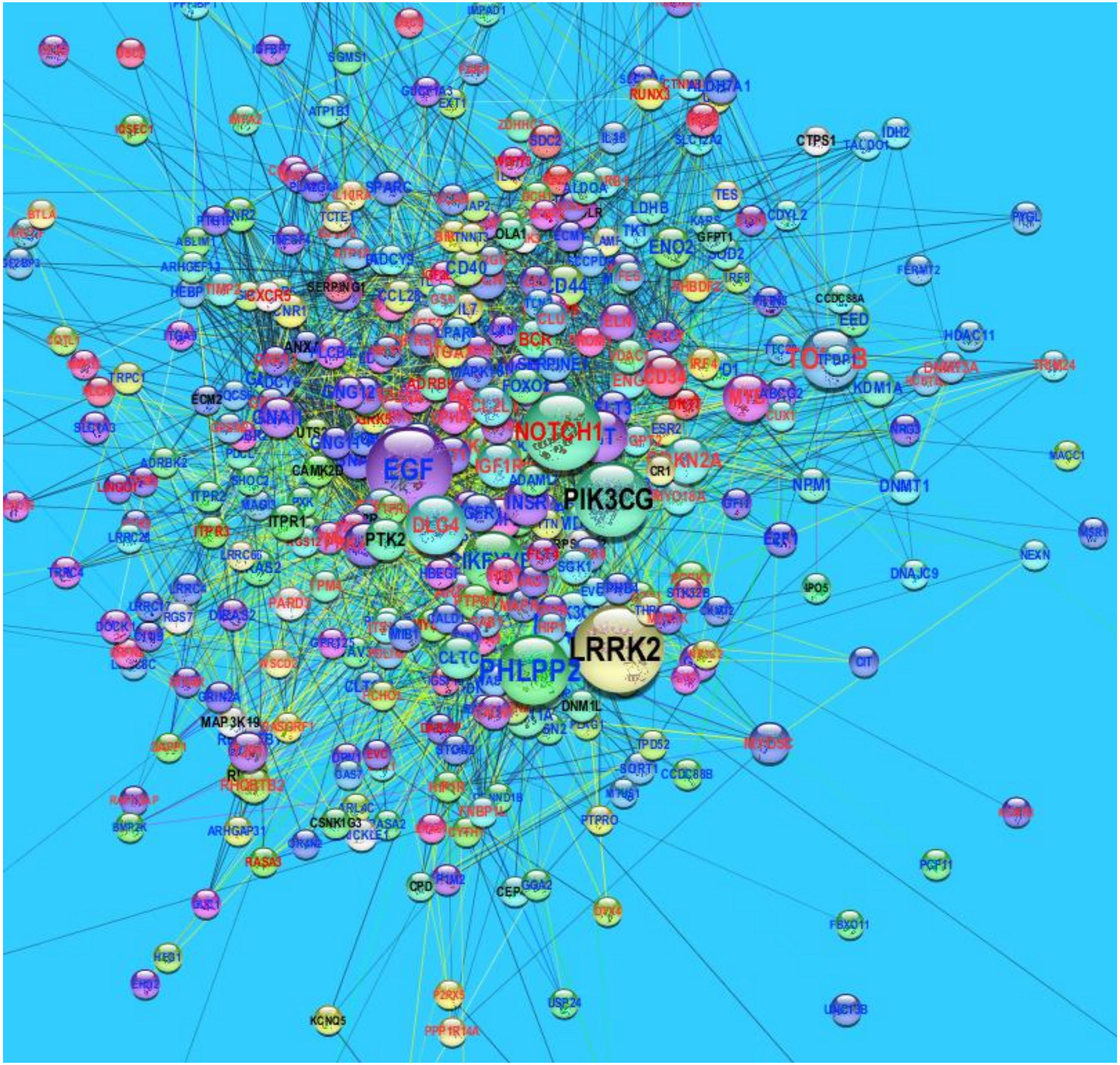

EGF
NOTCH1
PIK3CG
LRRK2
PHLPP2
DLG4
PTK2
INSR
CD44
CD40
CCL28
CXCR5
SERPING1
TIMP2
HEBP
FOXO1
GNAI1
CAMK2D
ITPR1
ITPR3
ITPR2
ECM2
TRPC1
SLC1A3
ADRBK2
SHOC2
PXK
LRRC28
RGS7
DIRAS2
PARD3
TPM4
GPR125
CLTC
FCHO1
GRIN2A
MAP3K19
RASGRF1
DNM1L
PLAG1
TPD52
SORT1
CCDC88B
PTPRO
CSNK1G3
ARHGAP31
RASA3
BMP2K
ARL4C
FNBP1L
CPD
GGA2
P2RX5
PPP1R14A
KCNQ5
USP24
HEG1
FBXO11
UNC13B
PCF11
CIT
DNAJC9
NEXN
MACC1
NPM1
DNMT1
HDAC11
KDM1A
DNMT3A
TRIM24
ABCG2
CUX1
ESR2
CR1
MYO18A
CDKN2A
EED
CCDC88A
FERMT2
PYGL
CDYL2
IDH2
TALDO1
CTPS1
SLC12A2
RUNX3
ALDH7A1
IL18
SDC2
LDHB
ALDOA
ENO2
TKT
GFPT1
SOD2
IRF8
RHBDF2
TES
SPARC
TCTE1
ABLIM1
ARHGEF12
BTLA
IQSEC1
ATP1B1
EXT1
SGMS1
IGFBP7
IMPAD1
BCR
ELN
CLU
IL7
VDAC1
IRF4
CD34
SGK1
STK32B
E2F1
GFI1
NRG3

Supplementary Figure S4. Sub-networks derived with K-means clustering from the subset of 285 network-related DMGs.

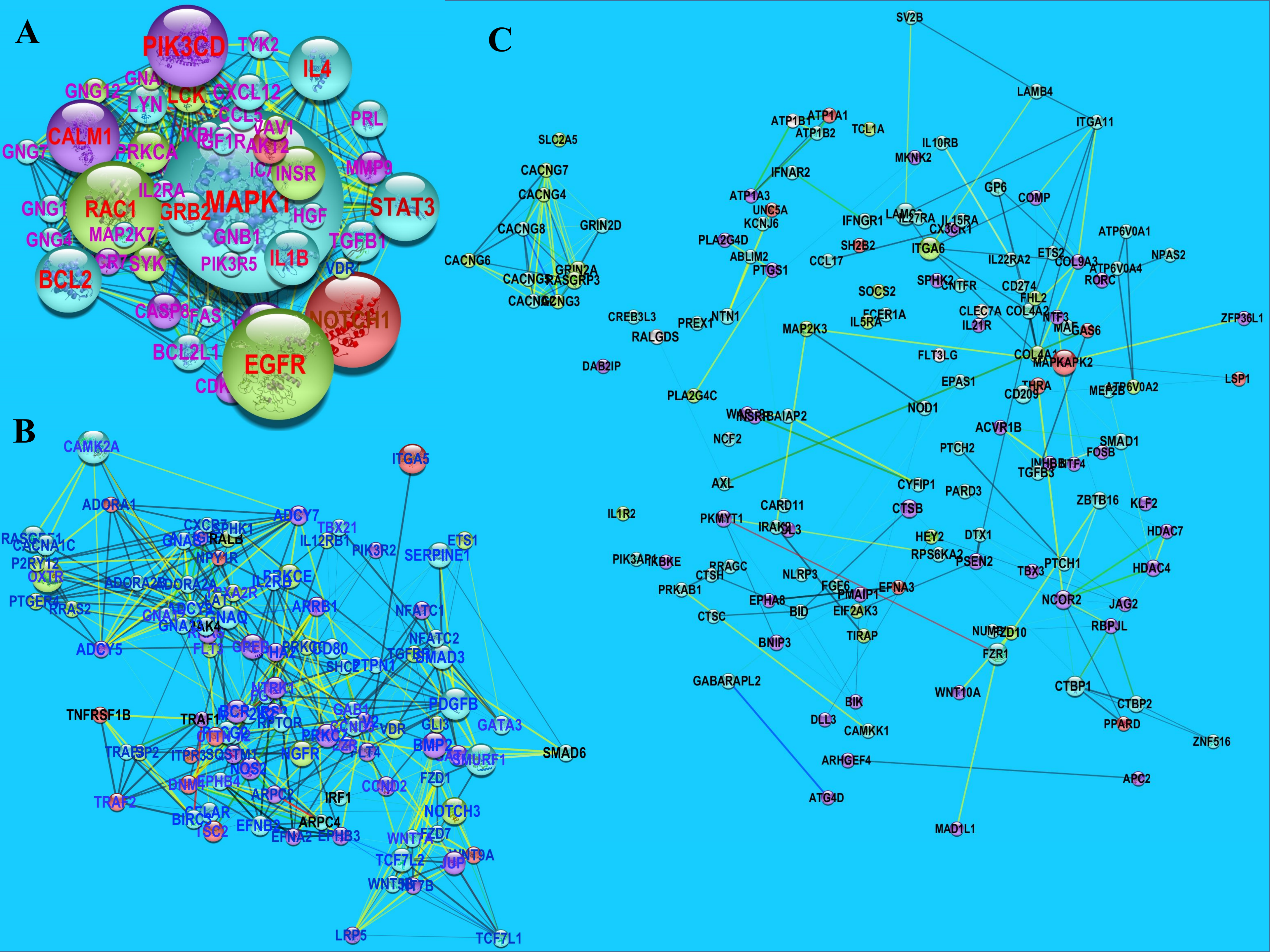
A
PIK3CD
TYK2
IL4
GNG12
LCK
CXCL12
LYN
CCL5
PRL
CALM1
VAV1
PRKCA
GNG7
INSR
MMP9
IL2RA
RAC1
GRB2
MAPK1
STAT3
GNG1
HGF
MAP2K7
GNG4
GNB1
TGFB1
SYK
PIK3R5
IL1B
VDR
BCL2
CASP8
FAS
NOTCH1
BCL2L1
EGFR
B
CAMK2A
ITGA5
ADORA1
ADCY7
TBX21
IL12RB1
PIK3R2
ETS1
SERPINE1
P2RY12
OXTR
ADCY5
NFATC1
NFATC2
SMAD3
TNFRSF1B
TRAF1
PDGFB
GATA3
SMAD6
NGFR
TRAF2
BMP2
SMURF1
NOS2
COND2
FZD1
IRF1
ARPC4
NOTCH3
TCF7L2
LRP5
TCF7L1
C
SV2B
LAMB4
ITGA11
ATP1A1
TCL1A
IL10RB
MKNK2
SLC2A5
CACNG7
CACNG4
IFNAR2
GP6
COMP
ATP1A3
UNC5A
KCNJ6
IFNGR1
CACNG8
GRIN2D
ATP6V0A1
NPAS2
PLA2G4D
SH2B2
ITGA6
CACNG6
ABLIM2
PTGS1
CCL17
IL22RA2
RORC
SOCS2
CD274
FHL2
CREB3L3
NTN1
RALGDS
PREX1
MAP2K3
IL5RA
IL21R
ZFP36L1
DAB2IP
FLT3LG
MAPKAPK2
LSP1
PLA2G4C
EPAS1
NOD1
ACVR1B
NCF2
PTCH2
SMAD1
FOSB
TGFB3
AXL
CYFIP1
PARD3
IL1R2
CARD11
CTSB
PKMYT1
ZBTB16
KLF2
HEY2
DTX1
HDAC7
RPS6KA2
PSEN2
TBX3
PTCH1
HDAC4
PRKAB1
NLRP3
EFNA3
NCOR2
JAG2
CTSC
EPHA8
BID
EIF2AK3
TIRAP
RBPJL
BNIP3
FZR1
GABARAPL2
WNT10A
CTBP1
CTBP2
BIK
PPARD
ZNF516
DLL3
CAMKK1
ARHGEF4
APC2
ATG4D
MAD1L1

Supplementary Figure S5. Network enrichment analysis on KEGG pathways for module derived with Cytoscape app MCODE from the PPI network of 1775 DEG-DMGs.

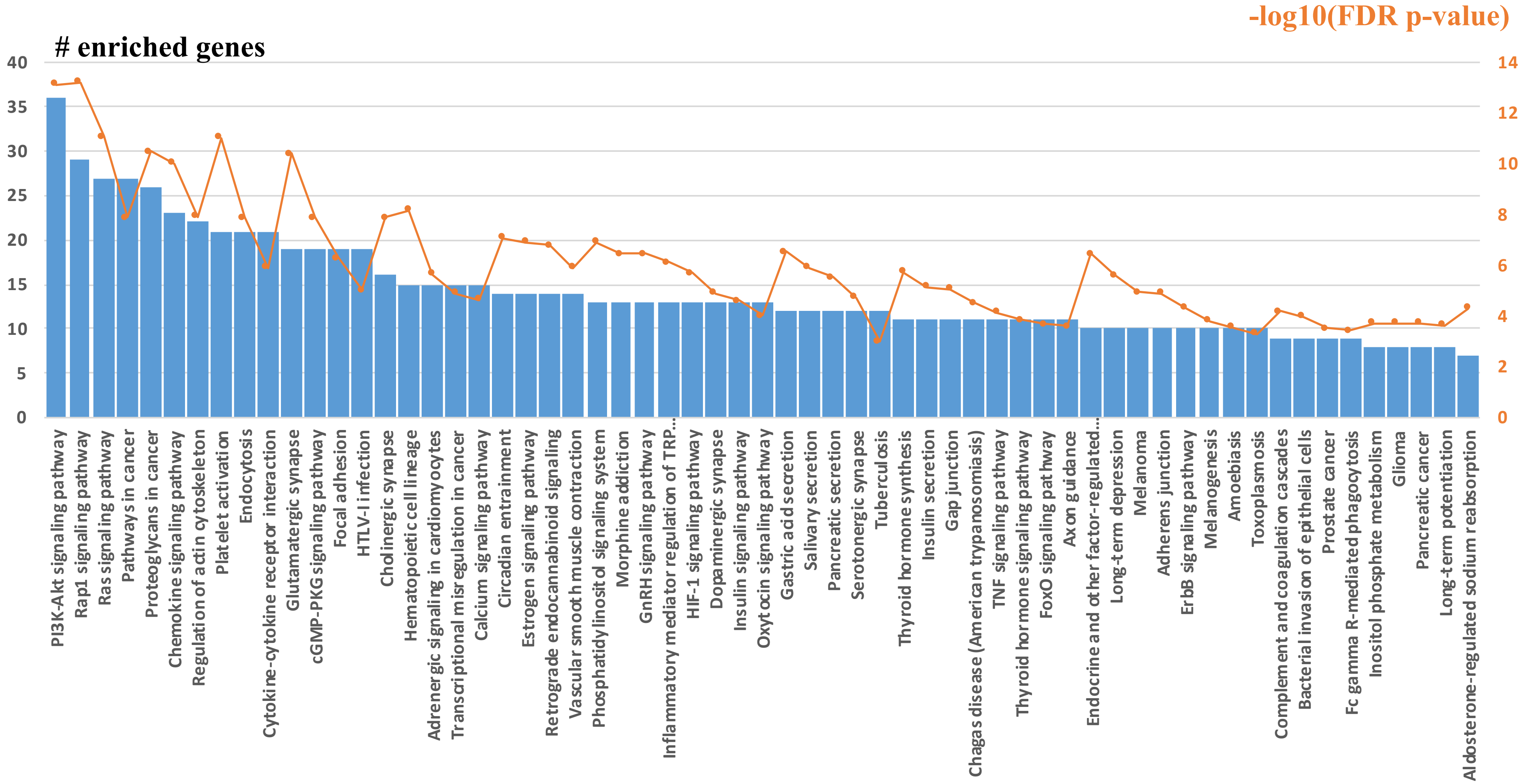

enriched genes
-log10(FDR p-value)
PI3K-Akt signaling pathway
Rap1 signaling pathway
Ras signaling pathway
Pathways in cancer
Proteoglycans in cancer
Chemokine signaling pathway
Regulation of actin cytoskeleton
Platelet activation
Endocytosis
Cytokine-cytokine receptor interaction
Glutamatergic synapse
cGMP-PKG signaling pathway
Focal adhesion
HTLV-I infection
Cholinergic synapse
Hematopoietic cell lineage
Adrenergic signaling in cardiomyocytes
Transcriptional misregulation in cancer
Calcium signaling pathway
Circadian entrainment
Estrogen signaling pathway
Retrograde endocannabinoid signaling
Vascular smooth muscle contraction
Phosphatidylinositol signaling system
Morphine addiction
GnRH signaling pathway
Inflammatory mediator regulation of TRP...
HIF-1 signaling pathway
Dopaminergic synapse
Insulin signaling pathway
Oxytocin signaling pathway
Gastric acid secretion
Salivary secretion
Pancreatic secretion
Serotonergic synapse
Tuberculosis
Thyroid hormone synthesis
Insulin secretion
Gap junction
Chagas disease (American trypanosomiasis)
TNF signaling pathway
Thyroid hormone signaling pathway
FoxO signaling pathway
Axon guidance
Endocrine and other factor-regulated...
Long-term depression
Melanoma
Adherens junction
ErbB signaling pathway
Melanogenesis
Amoebiasis
Toxoplasmosis
Complement and coagulation cascades
Bacterial invasion of epithelial cells
Prostate cancer
Fc gamma R-mediated phagocytosis
Inositol phosphate metabolism
Glioma
Pancreatic cancer
Long-term potentiation
Aldosterone-regulated sodium reabsorption

Supplementary Figure S6. PPI networks on the set of 191 network related DEG-DMGs. The PPI network was built with Cytoscape 11,12 from a subset of 191 DEG-DMGs previously obtained by applying network-based enrichment analysis 51. Nodes with the same color belong to the same cluster obtained by K-means clustering.

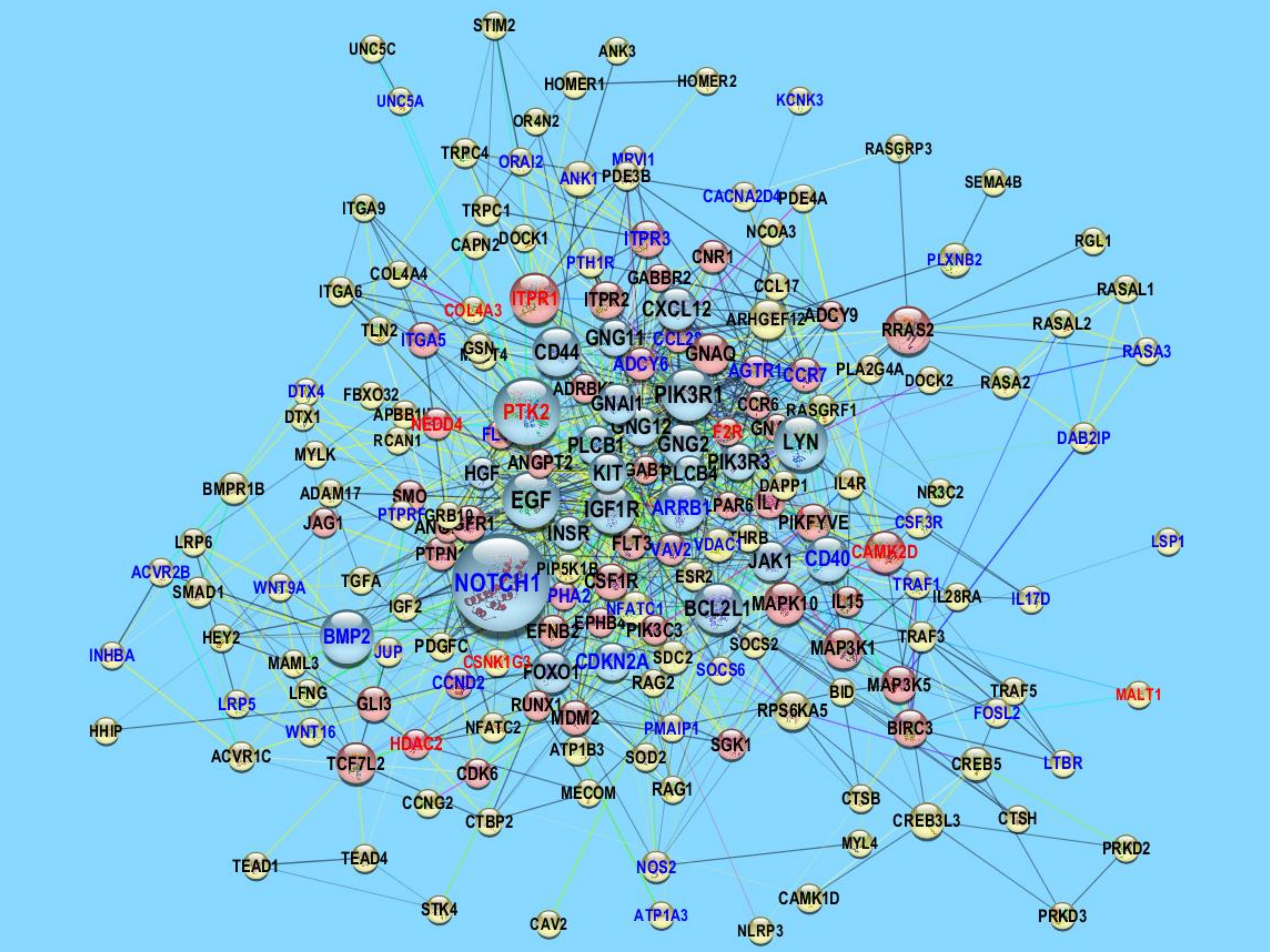

STIM2
UNC5C
ANK3
HOMER1
HOMER2
UNC5A
KCNK3
OR4N2
TRPC4
ORAI2
MRVI1
ANK1
PDE3B
RASGRP3
SEMA4B
CACNA2D4
PDE4A
ITGA9
TRPC1
NCOA3
CAPN2
DOCK1
ITPR3
CNR1
RGL1
PTH1R
PLXNB2
COL4A4
GABBR2
CCL17
ITGA6
ITPR1
ITPR2
RASAL1
COL4A3
CXCL12
ARHGEF12
ADCY9
RRAS2
RASAL2
TLN2
ITGA5
GNG11
GNAQ
CD44
RASA3
ADCY6
AGTR1
CCR7
PLA2G4A
DOCK2
RASA2
DTX4
FBXO32
PIK3R1
GNAI1
CCR6
RASGRF1
DTX1
PTK2
NEDD4
GNG12
LYN
RCAN1
F2R
GNG2
PLCB1
DAB2IP
MYLK
ANGPT2
PIK3R3
HGF
KIT
PLCB4
IL4R
BMPR1B
ADAM17
SMO
DAPP1
NR3C2
EGF
IGF1R
ARRB1
LPAR6
PIKFYVE
CSF3R
JAG1
PTPRF
GRB10
INSR
LRP6
FLT3
VAV2
VDAC1
THRB
LSP1
JAK1
CD40
CAMK2D
ACVR2B
TGFA
PIP5K1B
ESR2
WNT9A
SMAD1
NOTCH1
CSF1R
TRAF1
IGF2
NFATC1
BCL2L1
MAPK10
IL15
IL28RA
IL17D
HEY2
BMP2
EFNB2
PIK3C3
TRAF3
INHBA
JUP
PDGFC
SOCS2
MAP3K1
MAML3
CSNK1G3
CDKN2A
SDC2
FOXO1
SOCS6
CCND2
RAG2
MAP3K5
LFNG
BID
TRAF5
MALT1
LRP5
GLI3
RUNX1
MDM2
RPS6KA5
FOSL2
HHIP
WNT16
NFATC2
PMAIP1
BIRC3
HDAC2
SGK1
ACVR1C
TCF7L2
ATP1B3
SOD2
CREB5
LTBR
CDK6
CCNG2
MECOM
RAG1
CTSB
CTBP2
CREB3L3
CTSH
MYL4
PRKD2
TEAD4
TEAD1
NOS2
CAMK1D
STK4
CAV2
ATP1A3
NLRP3
PRKD3

Supplementary Figure S7. Association between methylation and gene expression. **A**, Spearman's rank correlation rho between variables for absolute value of logarithm base 2 of fold change (log_2FC) in gene expression at DEG-DMGs and the differences in methylation densities (between control and patient groups). All correlations are statistically significant (*p-value* lesser than 0.001). The variables analyzed are the absolute difference (Δ) of: $p_{density}$: density of methylation levels, $TV_{density}$: density of the difference of methylation levels, $TVB_{density}$: *TV* with Bayesian correction, and $HD_{density}$: density of Hellinger divergence of methylation levels. **B**, **D**, and **F** panels show two-dimensional kernel estimations (2D-KDE) of the joint probability distribution for each annotated pair of variables in the coordinate axes from the contour-plot plane (see main text for variable description). **C** and **E** panels: Farlie-Gumbel-Morgenstern (FGM) copula joint probability distribution built from the estimation of marginals distribution (*XZ* plane: Gamma probability distribution and *YZ* plane: generalized gamma distribution). Together, panels **A** to **F** indicate that, in the current study of patients with PALL, methylation and gene expression are not statistically independent, but associated with statistically highly significant linear trend, located with high joint probability in the outlined contour-plot red regions.

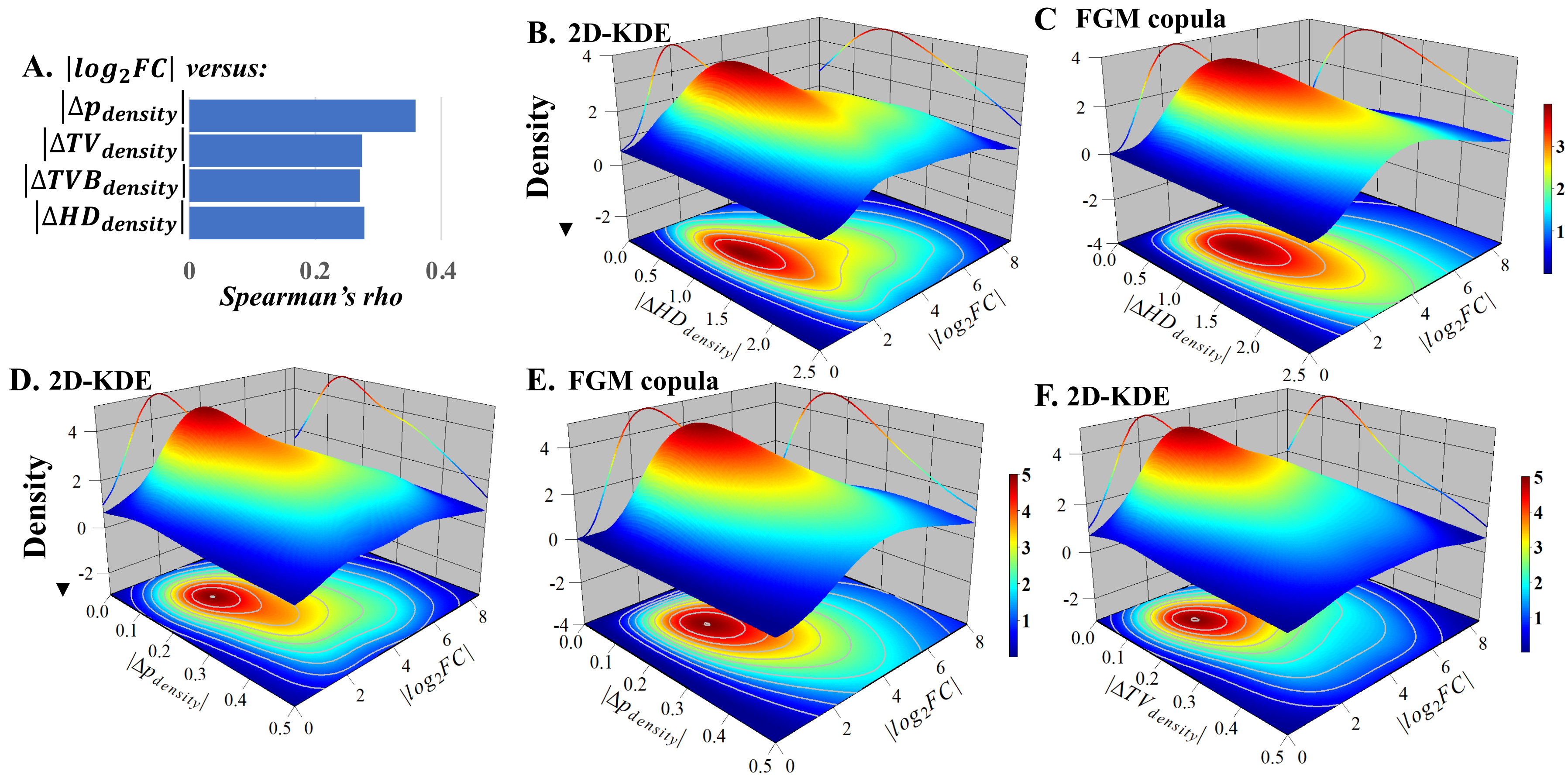

A. $|log_2FC|$ versus:
$|\Delta p_{density}|$
$|\Delta TV_{density}|$
$|\Delta TVB_{density}|$
$|\Delta HD_{density}|$
0
0.2
0.4
Spearman's rho
B. 2D-KDE
Density
$|\Delta HD_{density}|$
$|log_2FC|$
C FGM copula
$|\Delta HD_{density}|$
$|log_2FC|$
D. 2D-KDE
Density
$|\Delta p_{density}|$
$|log_2FC|$
E. FGM copula
$|\Delta p_{density}|$
$|log_2FC|$
F. 2D-KDE
$|\Delta TV_{density}|$
$|log_2FC|$

Supplementary Figure S8. Correlation analyses of the methylation signal on gene hubs across the individuals from control and patient groups. Heat-map showing the representation of network hubs as vectors of the methylation signals on genes across samples. DMG and DEG-DMG network hubs are clustered based on their correlation as distance measure (see Methods). Clusters of hubs are integrated by genes carrying highly correlated methylation signals.

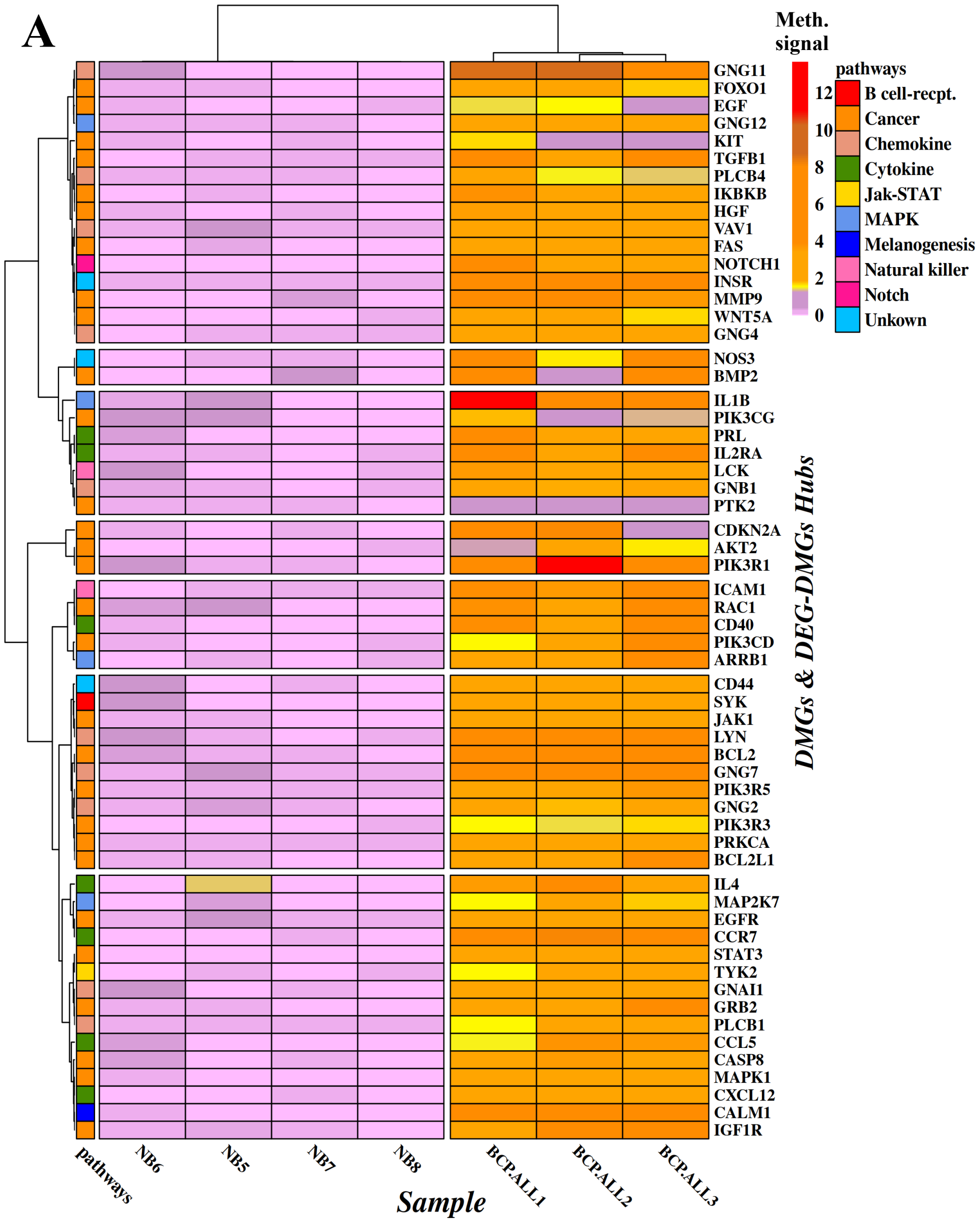

A
Meth. signal
12
10
8
6
4
2
0
pathways
B cell-recpt.
Cancer
Chemokine
Cytokine
Jak-STAT
MAPK
Melanogenesis
Natural killer
Notch
Unkown
GNG11
FOXO1
EGF
GNG12
KIT
TGFB1
PLCB4
IKBKB
HGF
VAV1
FAS
NOTCH1
INSR
MMP9
WNT5A
GNG4
NOS3
BMP2
IL1B
PIK3CG
PRL
IL2RA
LCK
GNB1
PTK2
CDKN2A
AKT2
PIK3R1
ICAM1
RAC1
CD40
PIK3CD
ARRB1
CD44
SYK
JAK1
LYN
BCL2
GNG7
PIK3R5
GNG2
PIK3R3
PRKCA
BCL2L1
IL4
MAP2K7
EGFR
CCR7
STAT3
TYK2
GNAI1
GRB2
PLCB1
CCL5
CASP8
MAPK1
CXCL12
CALM1
IGF1R
DMGs & DEG-DMGs Hubs
pathways
NB6
NB5
NB7
NB8
BCP.ALL1
BCP.ALL2
BCP.ALL3
Sample

Supplementary Figure S9. Heat-map for hub correlation matrix.

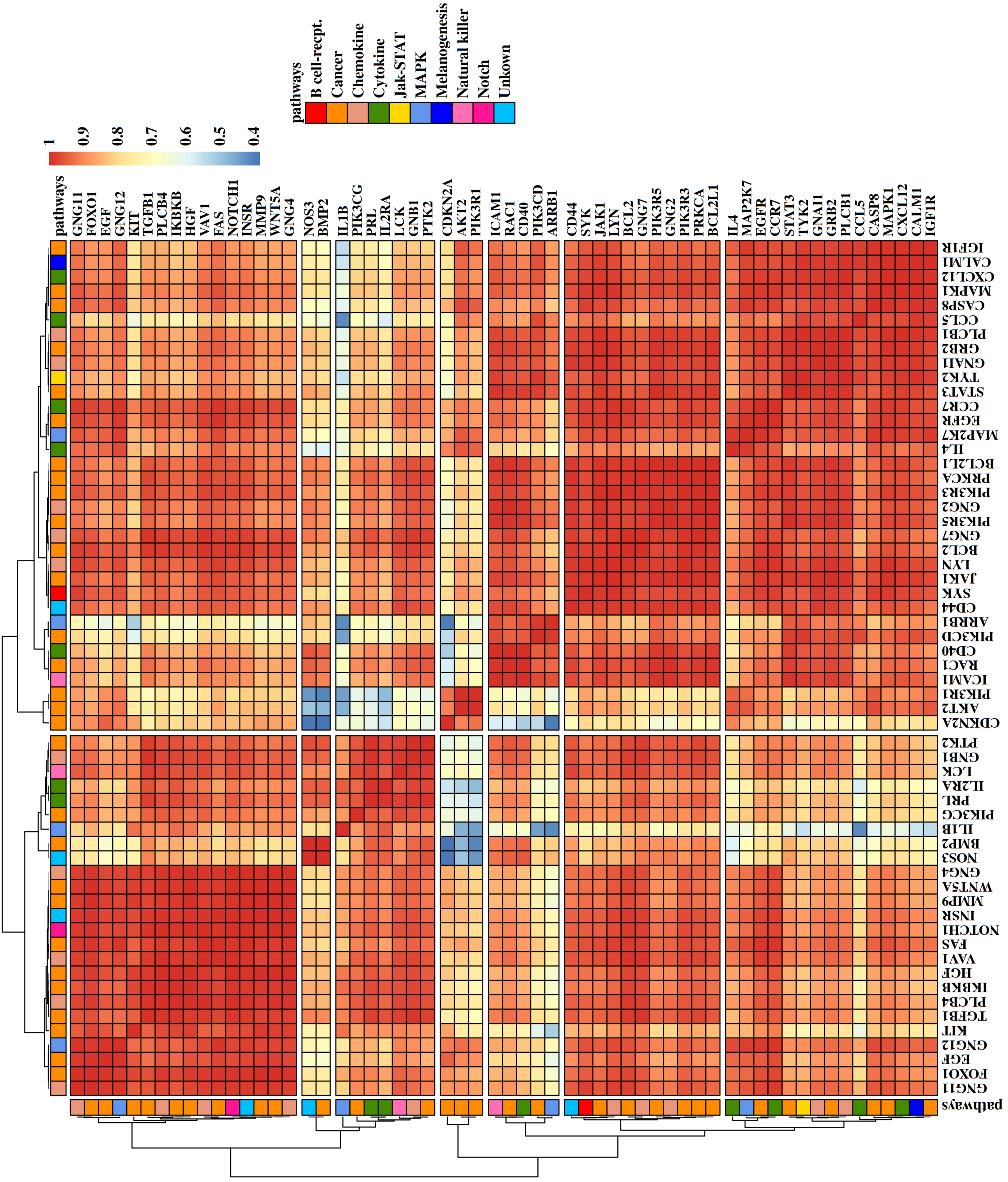

pathways
B cell-recept.
Cancer
Chemokine
Cytokine
Jak-STAT
MAPK
Melanogenesis
Natural killer
Notch
Unkown
1
0.9
0.8
0.7
0.6
0.5
0.4
GNG11
FOXO1
EGF
GNG12
KIT
TGFB1
PLCB4
IKBKB
HGF
VAV1
FAS
NOTCH1
INSR
MMP9
WNT5A
GNG4
NOS3
BMP2
IL1B
PIK3CG
PRL
IL2RA
LCK
GNB1
PTK2
CDKN2A
AKT2
PIK3R1
ICAM1
RAC1
CD40
PIK3CD
ARRB1
CD44
SYK
JAK1
LYN
BCL2
GNG7
PIK3R5
GNG2
PIK3R3
PRKCA
BCL2L1
IL4
MAP2K7
EGFR
CCR7
STAT3
TYK2
GNAI1
GRB2
PLCB1
CCL5
CASP8
MAPK1
CXCL12
CALM1
IGF1R

Supplementary Figure S10. PPI network on the set of DMG and DEG-DMGs hubs. The size of the nodes is proportional to the frequency of high-impact mutations, as reported in TCGA database.

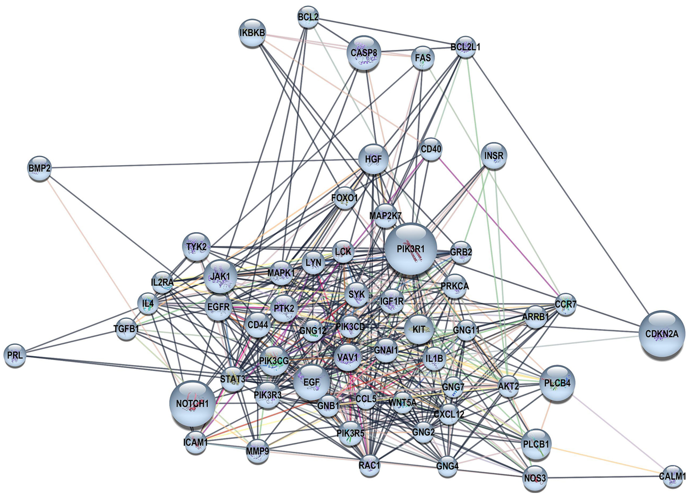
IKBKB
BCL2
CASP8
FAS
BCL2L1
BMP2
HGF
CD40
INSR
FOXO1
MAP2K7
TYK2
LCK
PIK3R1
GRB2
LYN
MAPK1
JAK1
IL2RA
SYK
IGF1R
PRKCA
IL4
EGFR
PTK2
CCR7
ARRB1
TGFB1
CD44
GNG12
PIK3CD
KIT
GNG11
CDKN2A
PRL
GNAI1
VAV1
IL1B
PIK3CG
STAT3
EGF
AKT2
PLCB4
GNG7
NOTCH1
PIK3R3
GNB1
CCL5
WNT5A
CXCL12
PIK3R5
GNG2
ICAM1
PLCB1
MMP9
RAC1
GNG4
NOS3
CALM1

Supplementary Figure S10. PPI network on the set of DMG and DEG-DMGs hubs. The size of the nodes is proportional to the frequency of high-impact mutations, as reported in TCGA database.

Integrative Network Analysis of Differentially Methylated and Expressed Genes for Biomarker Identification in Leukemia, R. Sanchez and S.A. Mackenzie

Supplementary information S1

Table of Contents

1 Cluster stability of PPI-networks partition into clusters 2

1.1 PPI-network of DMGs 2

1.2 PPI-network of DEGs-DMGs 3

2 Uncertainty in hierarchical cluster analysis 4

2.1 Cluster 1 8

2.2 Cluster 2 9

2.3 Cluster 3 10

3 Scaling of node degrees in PPI networks 10

4 Consistency of cluster mapping into PPI networks Consistency of cluster mapping into PPI networks 12

4.1.1 Auxiliary function to perform the p-value bootstrap estimation 13

4.2 Analysis for Cluster 1 14

4.2.1 Visualization of the network cohesive blocks in cluster 1 14

4.3 Analysis for Cluster 2 15

4.3.1 Visualization of the network cohesive blocks in cluster 2 16

4.4 Analysis for Cluster 3 16

4.4.1 Visualization of the network cohesive blocks in cluster 3 17

1 Cluster stability of PPI-networks partition into clusters

1.1 PPI-network of DMGs

The analysis of cluster stability obtained with K-means was performed with the R package *fpc* (version 2.2, Flexible Procedures for Clustering). The analysis for the K-mean partition of the PPI network of 285 DMGs into three clusters follows:

```
library(fpc)
### ==== The CSV file was created from the Supplementary Table S1 ====== ###

dmg.net <- read.csv("PPI-DMGs_network_statistics_(285_from_286_integrated_a_network).csv")
rownames(dmg.net) <- as.character(dmg.net$Alias)

### === Network centrality measures used in the analysis ===
dmg.net <- dmg.net[, c("BetweennessCentrality", "ClosenessCentrality",
                       "ClusteringCoefficient", "Degree")]
head(dmg.net)
#          BetweennessCentrality ClosenessCentrality ClusteringCoefficient Degree
# MAPK1              0.12768231           0.6042553             0.1643021    115
# EGFR               0.07275741           0.5843621             0.1970038     90
# NOTCH1             0.06100692           0.5419847             0.2064386     71
# RAC1               0.06021927           0.5308411             0.1855769     65
# PIK3CD             0.05031888           0.5772358             0.2250840     95
# CALM1              0.04356158           0.5399240             0.2333333     61

cl <- clusterboot(dmg.net, clustermethod = kmeansCBI, krange = 3, k = 3,
                  runs = 3, B = 10000, seed = 124 )
print(cl)
# * Cluster stability assessment *
#   Cluster method:  kmeans
# Full clustering results are given as parameter result
# of the clusterboot object, which also provides further statistics
# of the resampling results.
# Number of resampling runs:  10000
#
# Number of clusters found in data:  3
#
# Clusterwise Jaccard bootstrap (omitting multiple points) mean:
# [1] 0.8275131 0.9441247 0.8393312
# dissolved:
# [1] 1055    0  699
# recovered:
# [1] 6696 9837 7619
```

MANOVA test shows that differences between clusters are statistically significant.

```
grp = factor(cl$partition)
mova = manova( as.matrix(dmg.net) ~ grp )
summary(mova)
#             Df Pillai approx F num Df den Df    Pr(>F)
# grp          2 1.0892   83.709      8    560 < 2.2e-16 ***
#   Residuals 282
# ---
#   Signif. codes:  0 ‘***’ 0.001 ‘**’ 0.01 ‘*’ 0.05 ‘.’ 0.1 ‘ ’ 1
```

1.2 PPI-network of DEGs-DMGs

Next, the analysis for the K-mean partition of the PPI network of 191 DEGs-DMGs into three clusters follows:

```
### ==== The CSV file was created from the Supplementary Table S2 ====== ###

dmg.net <- read.csv("Statistics_PPI_network_of_191_DEGs-DMGs.csv")
rownames(dmg.net) <- as.character(dmg.net$Alias)

### === Network centrality measures used in the analysis ===
dmg.net <- dmg.net[, c("BetweennessCentrality", "ClosenessCentrality",
                       "ClusteringCoefficient", "Degree")]
head(dmg.net)
#        BetweennessCentrality ClosenessCentrality ClusteringCoefficient Degree
# UNC5C             0.00000000           0.3193277             1.0000000      2
# PMAIP1            0.00106971           0.4042553             0.6428571      8
# GNAI1             0.02266720           0.4523810             0.3809524     28
# HDAC2             0.00364942           0.3777336             0.2909091     11
# BIRC3             0.01669738           0.3869654             0.3717949     13
# GNG2              0.02652958           0.4702970             0.3598485     33

cl <- clusterboot(dmg.net, clustermethod = kmeansCBI, krange = 3, k = 3,
                  runs = 3, B = 10000, seed = 124 )
print(cl)
# * Cluster stability assessment *
# Cluster method:  kmeans
# Full clustering results are given as parameter result
# of the clusterboot object, which also provides further statistics
# of the resampling results.
# Number of resampling runs:  10000
#
# Number of clusters found in data:  3
#
# Clusterwise Jaccard bootstrap (omitting multiple points) mean:
# [1] 0.8650574 0.9637694 0.8539911
# dissolved:
# [1] 1339    0 1637
# recovered:
# [1] 8253 9987 8161
```

MANOVA test shows that differences between clusters are statistically significant.

```
grp = factor(cl$partition)
mova = manova( as.matrix(dmg.net) ~ grp )
summary(mova)
#             Df  Pillai approx F num Df den Df           Pr(>F)
# grp          2 0.95882   42.822             8    372 < 2.2e-16 ***
#   Residuals 188
# ---
#   Signif. codes:  0 ‘***’ 0.001 ‘**’ 0.01 ‘*’ 0.05 ‘.’ 0.1 ‘ ’ 1
```

2 Uncertainty in hierarchical cluster analysis

The uncertainty in hierarchical clusters from (main text Fig.9) was evaluated with the R package *pvclust* (version 2.0). For each cluster in hierarchical clustering, *p*-values were estimated via multiscale bootstrap resampling.
Function *pvclust* performs hierarchical cluster analysis via function hclust and automatically computes *p*-values for all clusters contained in the clustering of original data. It also provides graphical tools such as plot function or useful pvrect function which highlights clusters with relatively high/low p-values (see full descript at http://stat.sys.i.kyoto-u.ac.jp/prog/pvclust/).

Function *pvclust* provides two types of *p*-values: **AU** (**A**pproximately **U**nbiased) *p*-value and **BP** (**B**ootstrap **P**robability) value. **AU *p*-value, which is computed by multiscale bootstrap resampling, is a better approximation to unbiased *p*-value than BP** value computed by normal bootstrap resampling. Values on the edges of the clustering are *p*-values (%). Red values are **AU** *p*-values, and green values are **BP** values. Clusters with **AU** larger than 95% are highlighted by rectangles, which are strongly supported by data.

The datasets of genome-wide methylated and unmethylated read counts (for each cytosine site) from normal CD19+ blood cell donor (NB) and from patients with pediatric acute lymphoblastic leukemia (PALL) where downloaded from the Gene Expression Omnibus (GEO) database. DMPs were estimated for control (NB, GEO accession: GSM1978783 to GSM1978786) and for patients (ALL cells, GEO accession number GSM1978759 to GSM1978761) relative to a reference group of four independent normal CD19+ blood cell donor (GEO accession: GSM1978787 to GSM1978790).

Each individual, from control and patient groups, was represented by a vector of genes (networks hubs), where, in turns, each gene was represented by the numerical value of the density of Hellinger divergence per bp. The hierarchical clustering of DEG-DMGs from network and DMGs hubs is based on their Pearson correlation coefficient transformed to a dissimilarity measure: $\boldsymbol{dist}(x, y) = 1 - \boldsymbol{corr}(x, y)$, i.e., the distance $\boldsymbol{dist}(x, y)$ between genes *x* and *y* is a function of the correlation $\boldsymbol{corr}(x, y)$ between them. This information is visualized in the following heatmap (Supplementary Fig. S8):

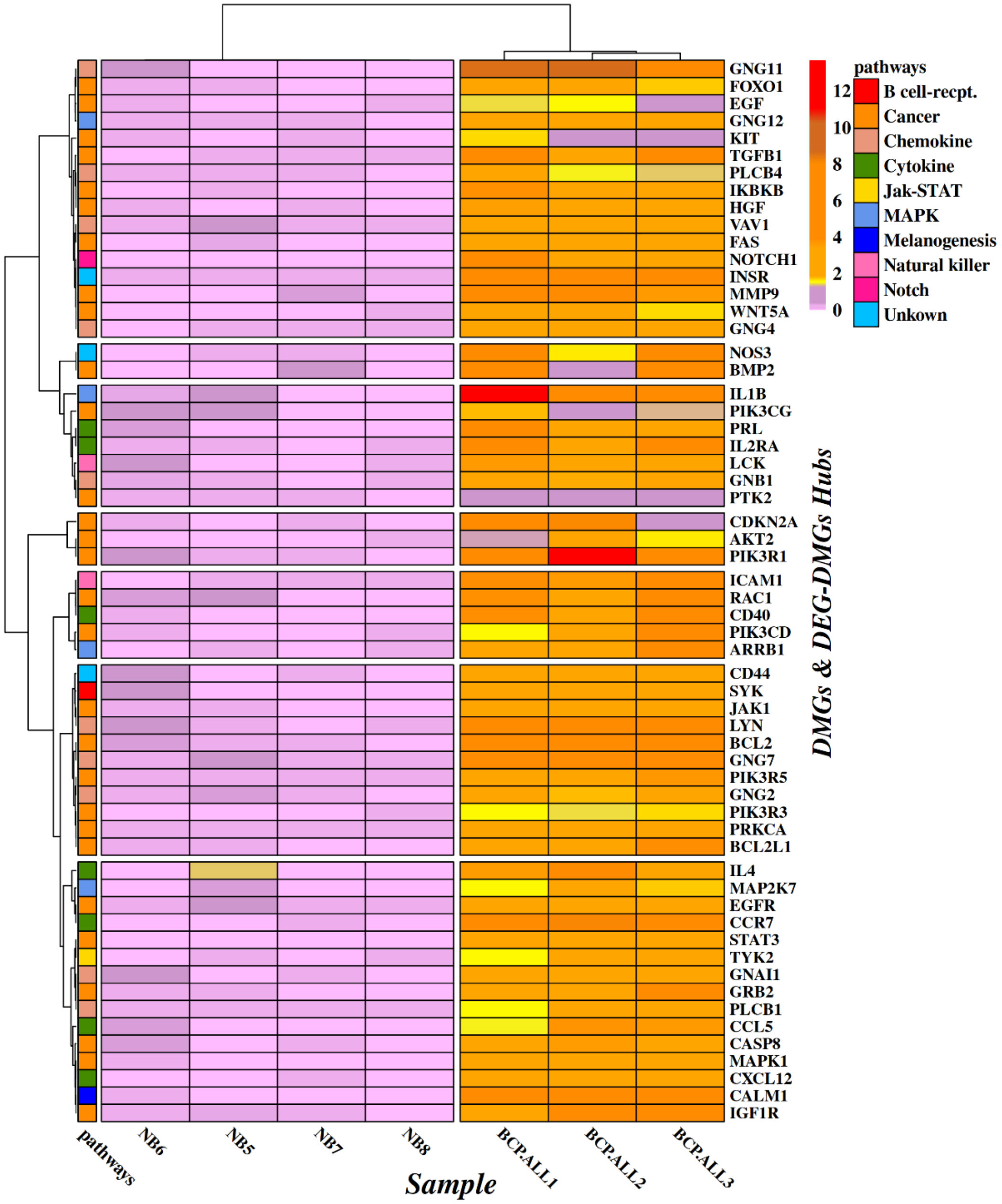

Then, the distance matrix used in the hierarchical cluster is derived from the corresponding correlation matrix:

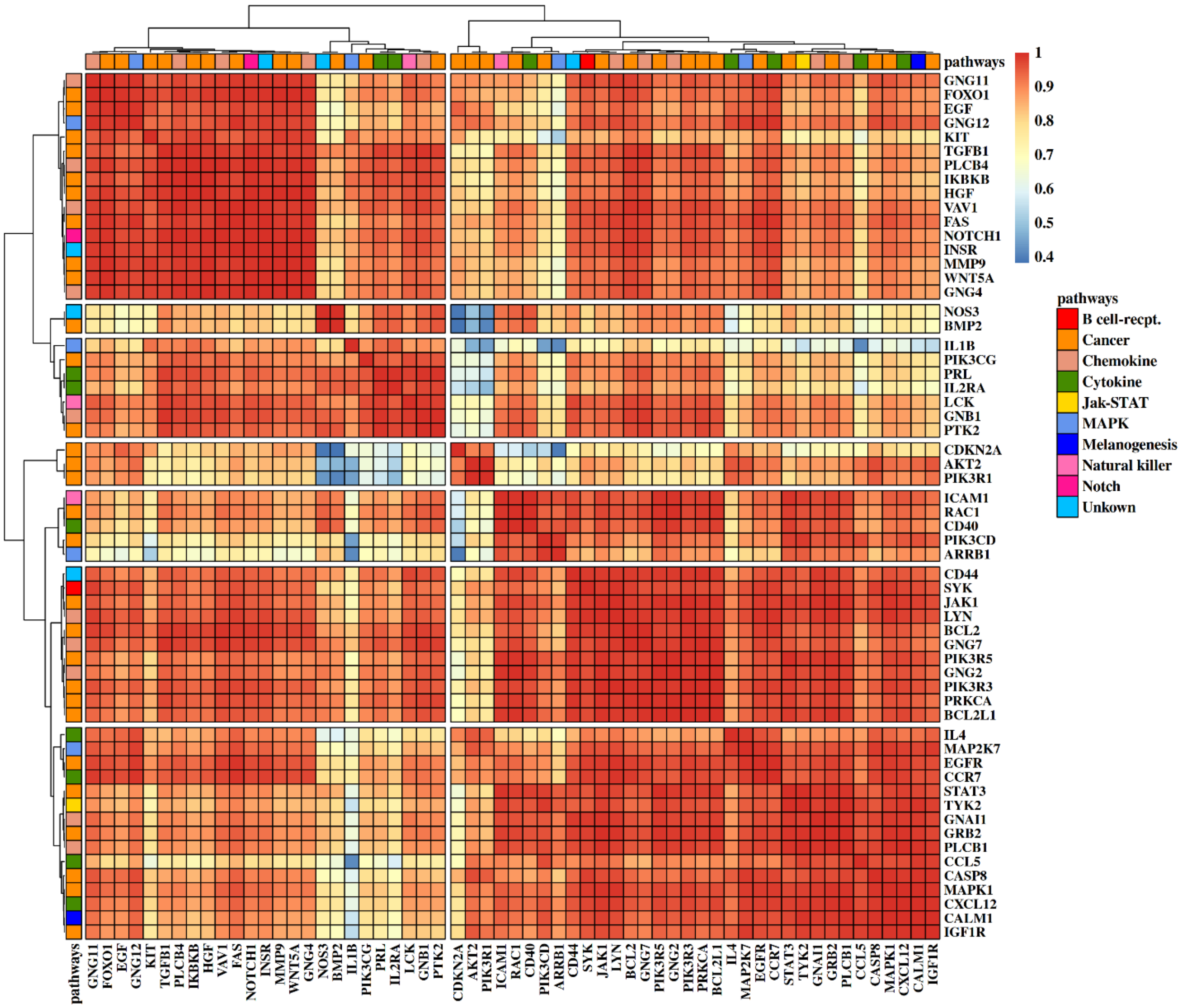

In above heat-map genes (hubs) are annotated according to KEEG pathway where they were primarily found. The color scale (blue (0.4) to red (close to 1)) denote the pairwise correlation between the corresponding genes.

To guide readers, the dendrogram from the hierarchical cluster, presented in Fig. 9A from main text, is given below:

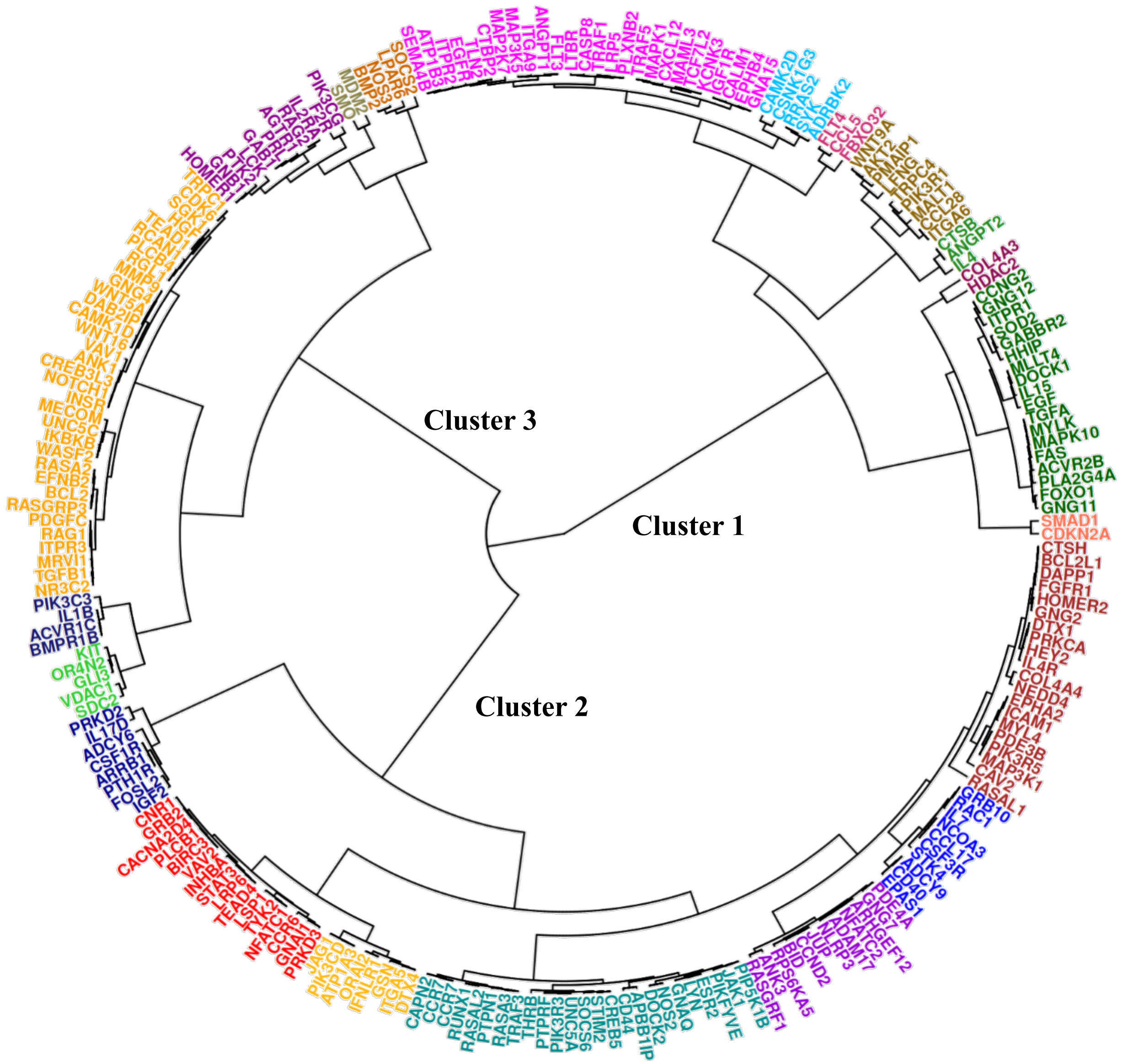
Cluster 3
Cluster 1
Cluster 2
SMAD1
CDKN2A
CTSH
BCL2L1
DAPP1
FGFR1
HOMER2
GNG2
DTX1
PRKCA
HEY2
RASGRP3
PDGFC
RAG1
ITPR3
MRVI1
TGFB1
NR3C2
PIK3C3
IL1B
ACVR1C
BMPR1B

2.1 Cluster 1

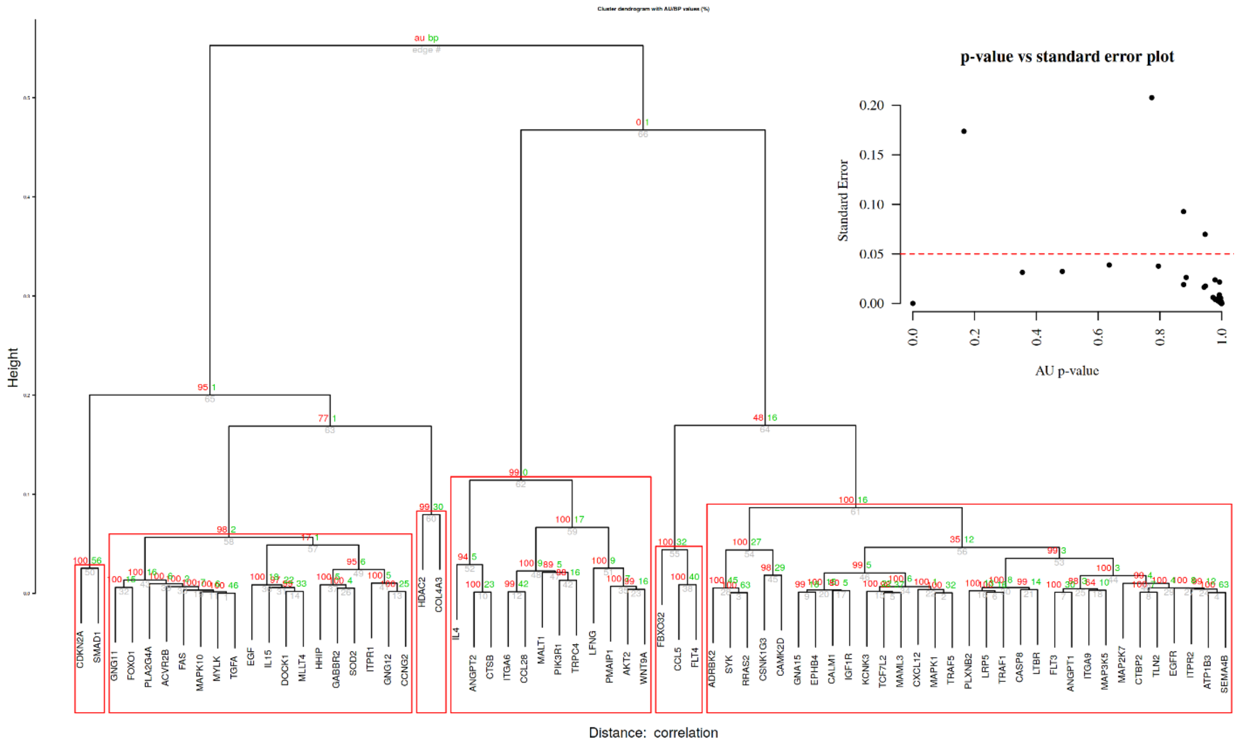

au bp
edge #
Height
Distance: correlation
CDKN2A
SMAD1
GNG11
FOXO1
PLA2G4A
ACVR2B
FAS
MAPK10
MYLK
TGFA
EGF
IL15
DOCK1
MLLT4
HHIP
GABBR2
SOD2
ITPR1
GNG12
CCNG2
HDAC2
COL4A3
IL4
ANGPT2
CTSB
ITGA6
CCL28
MALT1
PIK3R1
TRPC4
LFNG
PMAIP1
AKT2
WNT9A
FBXO32
CCL5
FLT4
ADRBK2
SYK
RRAS2
CSNK1G3
CAMK2D
GNA15
EPHB4
CALM1
IGF1R
KCNK3
TCF7L2
MAML3
CXCL12
MAPK1
TRAF5
PLXNB2
LRP5
TRAF1
CASP8
LTBR
FLT3
ANGPT1
ITGA9
MAP3K5
MAP2K7
CTBP2
TLN2
EGFR
ITPR2
ATP1B3
SEMA4B
p-value vs standard error plot
Standard Error
AU p-value
0.00
0.05
0.10
0.15
0.20
0.0
0.2
0.4
0.6
0.8
1.0

2.2 Cluster 2

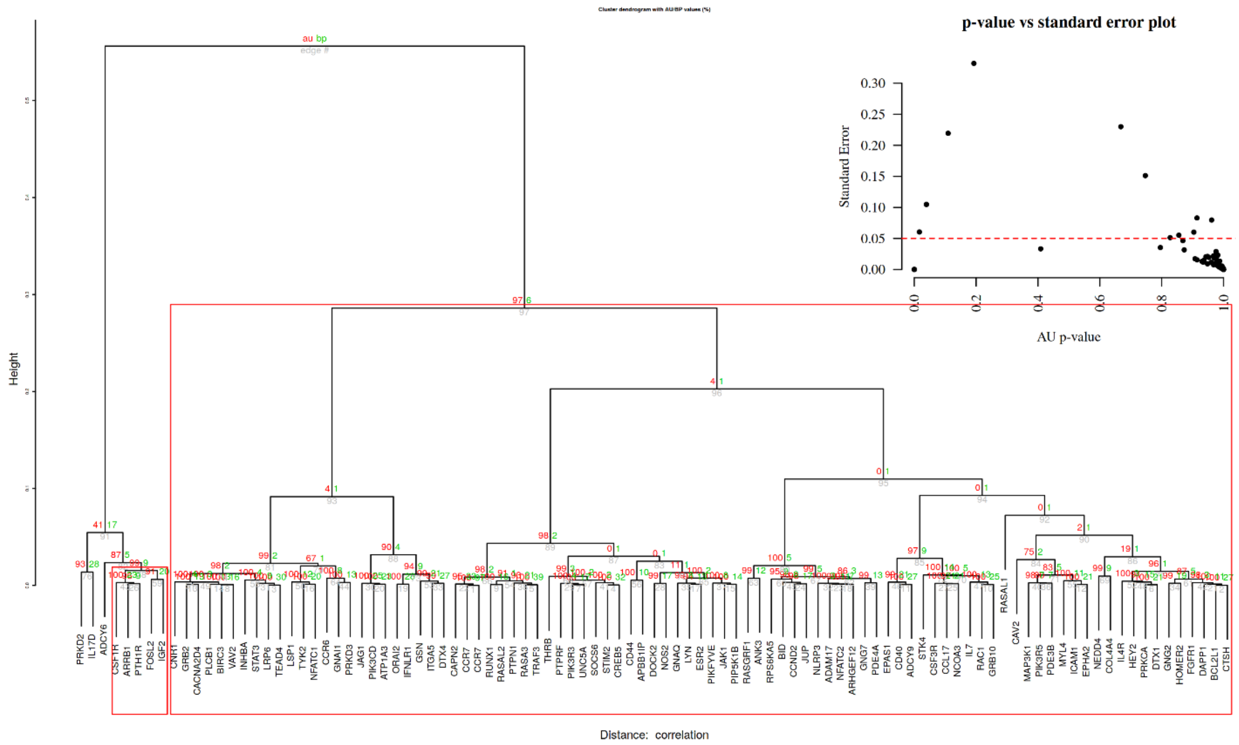

Cluster dendrogram with AU/BP values (%)
au bp
edge #
Height
Distance: correlation
p-value vs standard error plot
Standard Error
AU p-value

2.3 Cluster 3

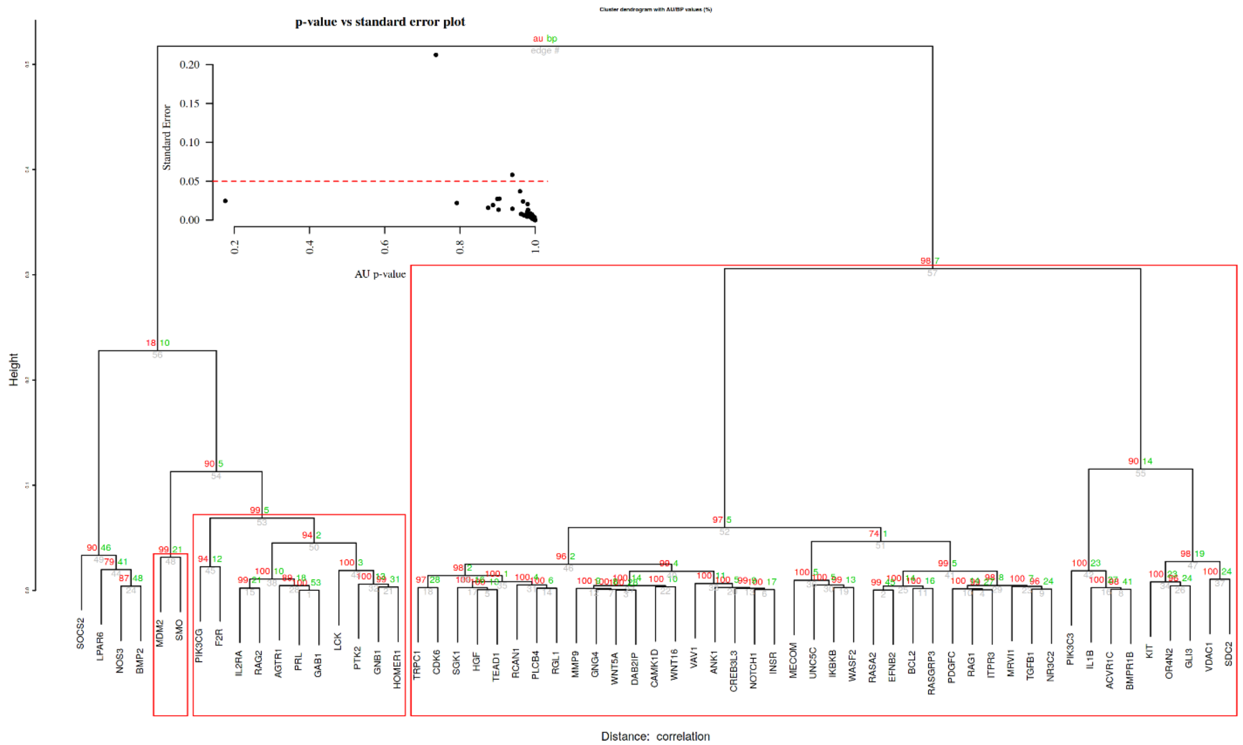

3 Scaling of node degrees in PPI networks

PPI networks with more than 100 genes are hard to visualize. Also, when applying network analysis, not all DMGs and DEGs estimated from the experimental datasets integrate networks. To work with subsets of the most relevant genes from the experimental dataset able to integrate networks is helpful to facilitate further network analysis. However, the estimated node centrality measures in the small network may depart from the truly the highest centrality values of the node in a larger network. We are confronting here a scaling issue.

A PPI network with 4378 DMGs (including DEGs-DMGs) was built. It turned out that there is a linear statistical trend between the node degree per total number of nodes (degree/#nodes) from the small network ($\boldsymbol{D_s}$) (285 DMGs plus 191 DEGs-DMGs) and the degree/#nodes from the

same DMGs in the large network (D_l). The figure below shows the results. The red-start points denote regression outliers (absolute value of the standardized residuals greater than 2)

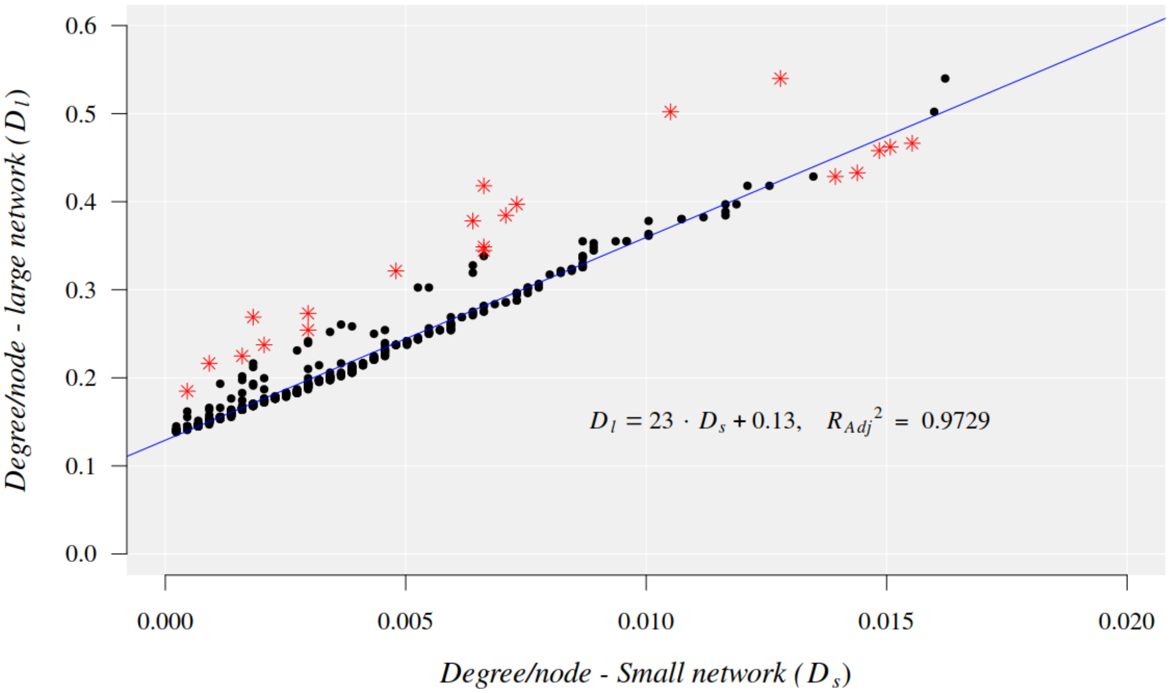

In other words, as a result of this linear statistical trend with pronounced positive slope, detected DMGs hubs from a small network will be hubs in larger networks as well. That is, the scaling preserves the hubs and smaller networks can be used to get a visual identification of hubs.

Centrality measures for the top twenty genes from the large network (sorted by their degree) are presented in the table below

Gene	Degree	Betweenness Centrality	Closeness Centrality	Clustering Coefficient
EGFR	459	0.042112	0.472682	0.061031
EGF	366	0.021668	0.454574	0.083569
MAPK1	341	0.024030	0.456102	0.076798
NOTCH1	336	0.019195	0.450472	0.089730
STAT3	308	0.014391	0.442937	0.103177
CCND1	257	0.010120	0.434648	0.103782
HDAC1	239	0.012427	0.423542	0.102317
RAC1	222	0.010386	0.422183	0.096572
PIK3R1	220	0.005951	0.423212	0.154379
CD44	218	0.006056	0.424411	0.147550
KIT	206	0.004863	0.421651	0.133602
GNB1	204	0.004140	0.405850	0.241572
IL4	204	0.005451	0.412715	0.159616
MMP9	203	0.006099	0.421528	0.133200
BMP4	199	0.006561	0.410763	0.128268
BRCA1	199	0.008751	0.415201	0.127912
IL1B	189	0.006933	0.414646	0.134245
GNG2	189	0.002982	0.401275	0.267984
CXCL12	185	0.002834	0.411035	0.243948
GRB2	183	0.005459	0.415002	0.127605

4 Consistency of cluster mapping into PPI networks Consistency of cluster mapping into PPI networks

Hub information from each cluster structure is integrated into a central cohesive block of the corresponding mapped network. The mapping of random gene subsets (sampled from the set DMGs hubs and the network DEGs-DMGs) into PPI networks restricted to nodes with 3 or more interactions and confidence interaction score of 0.7 (700), suggest that the hub-core information from each cluster structure is not random and statistically significant preserved in the PPI network. The probability to obtain similar mappings (from clusters to the cohesive block of hubs) just by random is lesser than 0.05.

The following R script can be run to reproduce the computations

To load the R packages and data

```
library(STRINGdb)
library(igraph)
```

```
##
## Attaching package: 'igraph'

## The following objects are masked from 'package:stats':
##
##     decompose, spectrum

## The following object is masked from 'package:base':
##
##     union

## --- To load the data ---
load(url("https://git.psu.edu/genomath/MethylIT_data/raw/master/pall_net-hubs-cluster-corr_10-
20-19.RData"))
```

Setting the interface to request for STRING human data on protein-protein interaction with confidence score of 0.7 (700):

```
string_db <- STRINGdb$new( version = "10", species = 9606, score_threshold = 700)
```

4.1.1 Auxiliary function to perform the *p*-value bootstrap estimation

```
pval <- function(geneSet, string_db, mapped, boots = 10, num.cores = 1L,
                 seed = 12, max.cohesion = 3) {

  set.seed(seed)
  l0 <- length(geneSet)
  l <- length(mapped$STRING_id)

  bootfun <- function(k) {
    y = data.frame(gene = geneSet[sample.int(l0, l)])
    mapped <- string_db$map( y, "gene", removeUnmappedRows = TRUE )
    grph <- string_db$get_subnetwork(mapped$STRING_id)
    blocks <- cohesive_blocks(grph)
    return(sum(max_cohesion(blocks) >= max.cohesion)/l)
  }

  grph <- string_db$get_subnetwork(mapped$STRING_id)
  blocks <- cohesive_blocks(grph)
  fraction_0 <- sum(max_cohesion(blocks) >= max.cohesion)/l0

  if (num.cores > 1) {
    require(BiocParallel)
    if (.Platform$OS.type == "unix") {
      bpparam <- MulticoreParam(workers=num.cores, tasks=0L,
                                progressbar = TRUE)
    } else bpparam <- SnowParam(workers=num.cores, progressbar = TRUE)
    fractions <- bplapply(seq_len(boots), function(k){
      x <- bootfun(k)
      return(x)}, BPPARAM=bpparam)
  } else {
    fractions <- vector(mode = "numeric", length = boots)
    for (k in seq_len(boots)) {
      cat("*** Performing boot ", k, " ...\n")
      fractions[k] <- bootfun(k)
    }
  }
  pval <- mean(c(fraction_0, fractions) >= fraction_0)
```

```
  return(list(p.value = pval, boots = c(fraction_0, fractions)))
}
```

Hierarchical cluster

```
hubs <- union(dmg.hubs, deg.dmg.hubs)
dmg_hubs_deg_dmg_net <- cbind(hubs., deg_dmg_net)
corr <- cor(x = dmg_hubs_deg_dmg_net)

hc = hclust(as.dist(2*(1 - corr)), method = "ward.D2")
grps = cutree(hc, k = 3 )
grp.names = names(grps) #
```

4.2 Analysis for Cluster 1

Requesting network information for DMGs from cluster 1

```
grp_1_nam = names(grps[grps == 1])
grp_1_hubs <- grp_1_nam[na.omit(match(hubs, grp_1_nam))]
g1_map_hubs <- string_db$map( data.frame(gene = grp_1_hubs), "gene",
                              removeUnmappedRows = TRUE )
```

Next, the bootstrapping p-value estimation is performed

```
pvals_hub_1 <- pval(geneSet = unique(c(dmg.hubs, deg.dmg.net)), string_db = string_db,
                    mapped = g1_map_hubs, boots = 5000, num.cores = 260L)

## Loading required package: BiocParallellibrary(STRINGdb)

  |=================================================================| 100%

grp_1_nam

##  [1] "MAPK1"   "EGFR"    "CALM1"   "IL4"     "AKT2"    "CASP8"   "SYK"
##  [8] "CCL5"    "MAP2K7"  "FAS"     "EGF"     "PIK3R1"  "CXCL12"  "IGF1R"
## [15] "GNG12"   "GNG11"   "CDKN2A"  "FOXO1"   "PMAIP1"  "HDAC2"   "DOCK1"
## [22] "GNA15"   "LRP5"    "CCNG2"   "HHIP"    "LTBR"    "CSNK1G3" "MALT1"
## [29] "ATP1B3"  "TRPC4"   "FLT4"    "FLT3"    "ITGA6"   "KCNK3"   "CAMK2D"
## [36] "SMAD1"   "CTBP2"   "MAPK10"  "TCF7L2"  "MYLK"    "WNT9A"   "ITGA9"
## [43] "ADRBK2"  "ANGPT1"  "ANGPT2"  "SEMA4B"  "GABBR2"  "COL4A3"  "CCL28"
## [50] "MLLT4"   "TGFA"    "FBXO32"  "ACVR2B"  "RRAS2"   "CTSB"    "MAP3K5"
## [57] "TRAF5"   "PLA2G4A" "TRAF1"   "EPHB4"   "SOD2"    "ITPR1"   "ITPR2"
## [64] "LFNG"    "PLXNB2"  "IL15"    "MAML3"   "TLN2"

pvals_hub_1$p.value

## [1] 0.01719656
```

4.2.1 Visualization of the network cohesive blocks in cluster 1

```
grph_1_hubs <- string_db$get_subnetwork(g1_map_hubs$STRING_id)
blocks_1_hubs <- cohesive_blocks(grph_1_hubs)

par(cex = 0.5)
plot(blocks_1_hubs, grph_1_hubs)
```

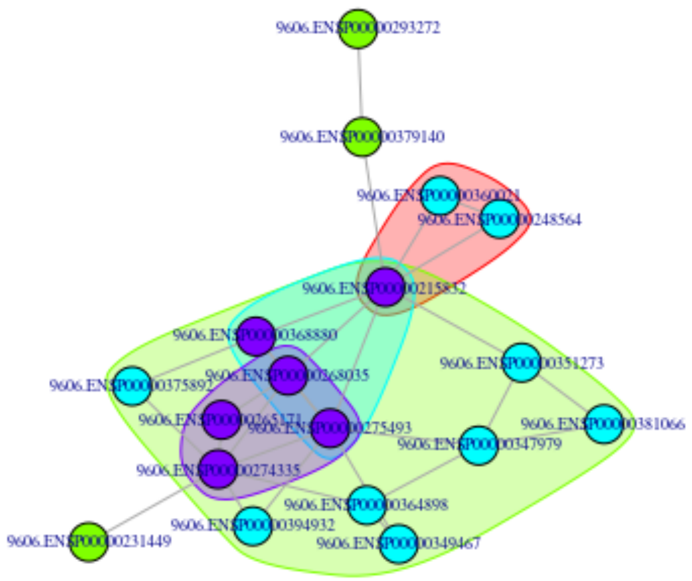

4.3 Analysis for Cluster 2

All the above sections are put together

```
grp_2_nam = names(grps[grps == 2])
grp_2_hubs <- grp_2_nam[na.omit(match(hubs, grp_2_nam))]
g2_map_hubs <- string_db$map( data.frame(gene = grp_2_hubs), "gene",
                              removeUnmappedRows = TRUE )

pvals_hub_2 <- pval(geneSet = unique(c(dmg.hubs, deg.dmg.net)), string_db = string_db,
                    mapped = g2_map_hubs, boots = 5000, num.cores = 20L)
```

```
  |=================================================================| 100%

grp_2_nam

##  [1] "RAC1"     "PIK3CD"   "STAT3"    "PRKCA"    "GRB2"     "TYK2"
##  [7] "CCR7"     "ICAM1"    "GNG7"     "PIK3R5"   "JAK1"     "PLCB1"
## [13] "GNAI1"    "GNG2"     "PIK3R3"   "CD40"     "BCL2L1"   "CD44"
## [19] "ARRB1"    "LYN"      "BIRC3"    "CSF1R"    "GRB10"    "DOCK2"
## [25] "LRP6"     "LSP1"     "INHBA"    "GNAQ"     "CSF3R"    "RASGRF1"
## [31] "PDE3B"    "PDE4A"    "ADCY6"    "ATP1A3"   "ADCY9"    "STIM2"
## [37] "NOS2"     "CCL17"    "NEDD4"    "CCR7"     "CCR6"     "NCOA3"
## [43] "ESR2"     "RASAL1"   "CAPN2"    "MYL4"     "DAPP1"    "TEAD4"
## [49] "NFATC1"   "NFATC2"   "ARHGEF12" "NLRP3"    "ITGA5"    "IL7"
## [55] "ANK3"     "CNR1"     "HEY2"     "IL4R"     "FOSL2"    "SOCS6"
## [61] "PTH1R"    "COL4A4"   "DTX4"     "CAV2"     "HOMER2"   "PTPN1"
## [67] "JAG1"     "FGFR1"    "RASA3"    "PIKFYVE"  "RUNX1"    "DTX1"
## [73] "UNC5A"    "EPHA2"    "GSN"      "CREB5"    "PRKD3"    "RASAL2"
## [79] "PRKD2"    "IGF2"     "BID"      "ADAM17"   "APBB1IP"  "PTPRF"
## [85] "ORAI2"    "MAP3K1"   "TRAF3"    "THRB"     "PIP5K1B"  "CTSH"
## [91] "CCND2"    "RPS6KA5"  "IL17D"    "STK4"     "VAV2"     "IFNLR1"
## [97] "CACNA2D4" "JUP"      "EPAS1"

pvals_hub_2$p.value
```

```
## [1] 0.03059388
```

4.3.1 Visualization of the network cohesive blocks in cluster 2

```
grph_2_hubs <- string_db$get_subnetwork(g2_map_hubs$STRING_id)
blocks_2_hubs <- cohesive_blocks(grph_2_hubs)

par(cex = 0.5)
plot(blocks_2_hubs, grph_2_hubs)
```

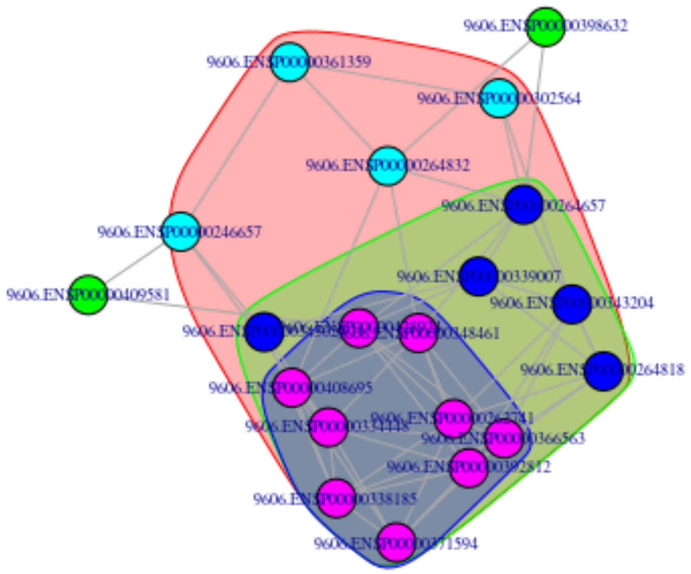

4.4 Analysis for Cluster 3

```
grp_3_nam = names(grps[grps == 3])
grp_3_hubs <- grp_3_nam[na.omit(match(hubs, grp_3_nam))]
g3_map_hubs <- string_db$map( data.frame(gene = grp_3_hubs), "gene",
                              removeUnmappedRows = TRUE )

pvals_hub_3 <- pval(geneSet = unique(c(dmg.hubs, deg.dmg.net)), string_db = string_db,
                    mapped = g3_map_hubs, boots = 5000, num.cores = 20L)

  |=================================================================| 100%

grp_3_nam

##  [1] "BCL2"    "IL1B"    "WNT5A"   "TGFB1"   "PRL"     "LCK"     "MMP9"
##  [8] "GNB1"    "IKBKB"   "NOS3"    "VAV1"    "GNG4"    "IL2RA"   "NOTCH1"
## [15] "INSR"    "PLCB4"   "PIK3CG"  "HGF"     "BMP2"    "PTK2"    "KIT"
## [22] "UNC5C"   "SGK1"    "VDAC1"   "RASA2"   "GAB1"    "RGL1"    "TRPC1"
## [29] "PIK3C3"  "MDM2"    "SDC2"    "CREB3L3" "TEAD1"   "CDK6"    "RASGRP3"
## [36] "MECOM"   "ANK1"    "RCAN1"   "EFNB2"   "SMO"     "NR3C2"   "LPAR6"
## [43] "HOMER1"  "AGTR1"   "OR4N2"   "DAB2IP"  "ACVR1C"  "F2R"     "RAG1"
```

```
## [50] "RAG2"    "CAMK1D"  "WNT16"   "PDGFC"   "BMPR1B"  "SOCS2"   "GLI3"
## [57] "ITPR3"   "MRVI1"   "WASF2"

pvals_hub_3$p.value

## [1] 0.03379324
```

4.4.1 Visualization of the network cohesive blocks in cluster 3

```
grph_3_hubs <- string_db$get_subnetwork(g3_map_hubs$STRING_id)
blocks_3_hubs <- cohesive_blocks(grph_3_hubs)

par(cex = 0.5)
plot(blocks_3_hubs, grph_3_hubs)
```

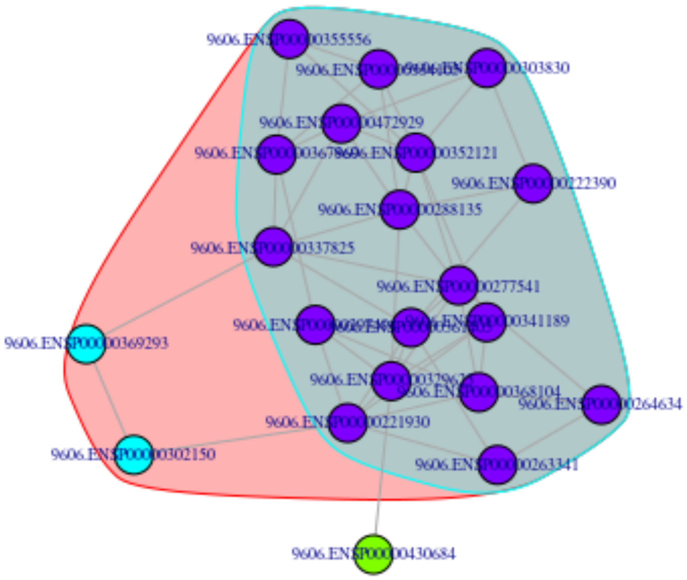

Supplement: Supplementary file 1 — Supplementary Figures and Information. [file 41598_2020_58123_MOESM1_ESM.pdf]
